# Atomistic scattering modeling of the solution structure of human dimeric IgA1 reveals a structural and mechanistic basis for IgA nephropathy

**DOI:** 10.1016/j.jbc.2026.113156

**Published:** 2026-05-14

**Authors:** Jayesh S. Bhatt, See Cheng Yeo, Sam M. Ireland, Aisha Ben-Younis, Jayesh Gor, Karen Molyneux, Jonathan Barratt, Stephen J. Perkins

**Affiliations:** 1 Division of Biosciences, Department of Structural and Molecular Biology, University College London, London, United Kingdom; 2 Department of Cardiovascular Medicine, University of Leicester, Leicester, United Kingdom

**Keywords:** analytical ultracentrifugation, antibody, atomistic modeling, IgA1 dimer, molecular dynamics, small-angle neutron scattering, small-angle X-ray scattering

## Abstract

IgA nephropathy (IgAN) is characterized by mesangial deposition of J-chain containing dimeric IgA1 (dIgA1). Given that deposited IgA1 is mostly dIgA1, solution structure determinations of dIgA1 in phenotyped patients may be relevant to the pathogenesis of IgAN. Thus, dIgA1 from three IgAN patients and a healthy control was isolated. Biochemically, the dIgA1 structures were correlated with serum IgA–IgG immune complex levels, activated human mesangial cells interleukin-6 production, and the pattern of IgA1 *N*- and *O*-glycosylation. The degree of dIgA1 hinge *O*-glycosylation varied between the four subjects, being lower in IgAN subjects with an active renal biopsy Oxford score and clinically progressive IgAN. Their solution structures were investigated using analytical ultracentrifugation and small angle X-ray and neutron scattering. The increased *O*-glycosylation of the dIgA1 hinge was associated with larger X-ray radii of gyration $R_{g}^{0}$ (healthy control, 8.75 ± 0.05 nm; patient A, 8.90±0.06 nm) compared to subjects with reduced *O-*glycosylation that showed more compact structures (patient B, 8.62±0.05 nm and patient C, 8.62±0.07 nm). Similar findings were observed by neutron scattering. Atomistic modeling of the X-ray data identified 100 best-fit structures that accounted for the scattering curves for each of the four dIgA1 samples. Analysis of these structures showed that the angle between the main axes of the two monomers in the dimeric structures for the three patients is slightly reduced compared to that of the healthy control. This difference implicates conformational change in the overall dIgA1 structure that may increase mesangial IgA deposition and ability to generate renal injury.

Immunoglobulin A (IgA) is the most abundant human immunoglobulin found on mucosal surfaces and the second most abundant in plasma (1, 2, 3, 4). It acts as a first line of defense against many pathogens, preventing their entry into the body. IgA antibodies can be divided into the IgA1 and IgA2 subclasses that are found in similar amounts on mucosal surfaces. IgA1 is characterized by extensive glycosylation in its hinge region. Dimeric IgA consists of two IgA monomers connected by a 15 kDa joining chain (J chain) at the bases of the Fc regions (Fig. 1), and the dimerization process involves the formation of disulfide bridges between two IgA monomers. Secretory IgA is formed when dimeric IgA binds to the polymeric immunoglobulin receptor expressed on the basolateral surface of mucosal epithelial cells. Following binding, the IgA-receptor complex is transcytosed across the epithelial cell for secretion into the mucosal lumen. During transcytosis, the polymeric immunoglobulin receptor is proteolytically cleaved with a portion of the receptor, secretory component, covalently binding to IgA, forming secretory IgA. Secretory component protects the IgA from proteolytic degradation by luminal bacterial proteases.

**Figure 1:**
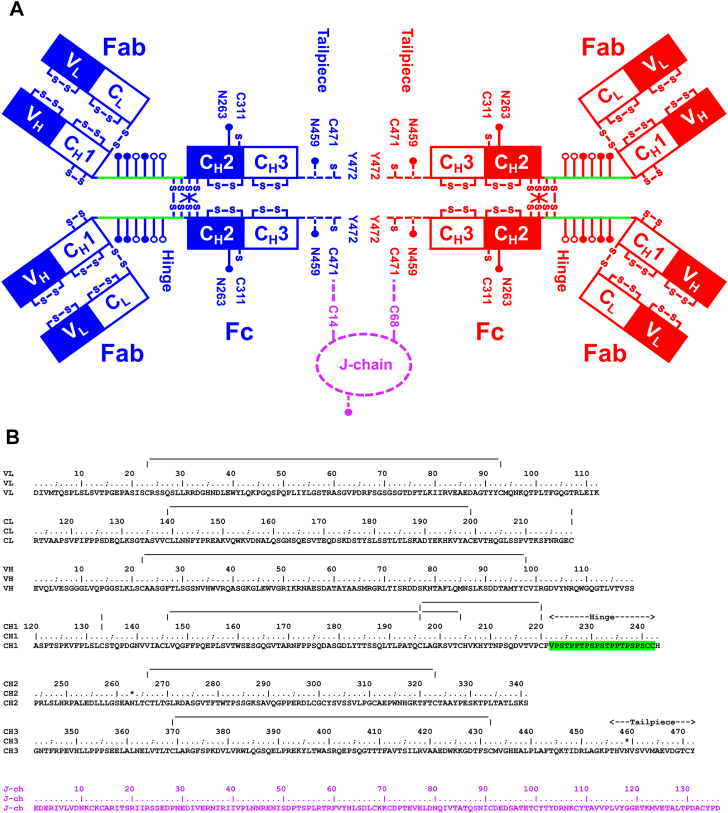
**Domain arrangement and sequence of dIgA1**. *A*, the schematic cartoon shows two IgA1 monomers (*red* and *blue*) connected by a smaller J-chain (*purple*). Each monomer contains two heavy chains with variable domain (V_H_) and constant domains (C_H_1, C_H_2, and C_H_3) plus two light chains with V_L_ and C_L_ domains. Interchain disulfide bridges, indicated with the letter ‘S’, stabilize the IgA1 structure. Two conserved N-glycosylation sites occurred at Asn^263^ and Asn^459^ (•). The hinge region between the Fab and Fc regions, highlighted with *solid green line*, is composed of 21 residues (222-PVPSTPPTPSPSTPPTPSPSCC-242, anchored at Pro^221^ and His^243^), with the capacity to bind six O-linked glycans on each hinge (○ and •). The sites at Thr^225^, Thr^228^, and Ser^232^ were assumed to be occupied for the present study (•; Experimental). An 18-residue tailpiece between Pro^455^ and Tyr^472^ (*broken green line*) lies at the C terminus of each heavy chain. *B*, the sequence of human IgA1 monomer along with that of the J-chain is shown. Known disulfide bridges are indicated by vertical and horizontal lines. Asn^263^ and Asn^459^ are *asterisked*. dIgA1, dimeric IgA1; IgA1, immunoglobulin A1.

IgA nephropathy (IgAN) is the most common pattern of primary glomerulonephritis worldwide and remains an important cause of chronic kidney disease and kidney failure (5). Its hallmark is the mesangial deposition of J chain-containing dimeric IgA1 (dIgA1), which is often accompanied by mesangial cell proliferation and extracellular matrix production. It is widely postulated that the IgA deposited within the kidney is of systemic origin (6). This is supported, first, by the recurrence of IgAN in transplanted kidneys of patients with IgAN (7, 8), and second, the resolution of IgA deposits in transplanted kidneys when donor kidneys with IgA deposits were inadvertently transplanted to recipients without IgAN (9, 10). IgAN is thought to be the result of a four-hit process. This is initiated by the synthesis of IgA1 with a poorly *O*-galactosylated hinge region by a subpopulation of IgA1-secreting B cells (“first hit”). The “second hit” is the production of antiglycan antibodies which recognize the poorly galactosylated IgA1 hinge region. “Hit three” in this process is the formation of immune complexes, and “hit four” is the deposition of these immune complexes in the glomerular mesangium with triggering of glomerulonephritis. Protein structures determine the antigenic epitopes in a number of autoimmune kidney diseases, including antiglomerular basement membrane disease, antineutrophil cytoplasmic antibody-associated vasculitis, and primary membranous nephropathy (11, 12, 13). However, a role for conformational changes in dIgA1 and the development of IgAN remains unknown up to now. Such conformational changes could alter the interaction of dIgA1 with itself (*e.g.,* promoting self-aggregation) (14) or cell surface receptors (*e.g.,* promoting cell activation) (15, 16, 17, 18), extracellular matrix components (*e.g.,* promoting glomerular deposition) (14, 19), and other immunoglobulins (exposure of neo-antigenic epitopes for autoantibody formation) (20).

Protein structural studies of dIgA1 are made difficult due to its large multidomain structure with *O-*linked and *N-*linked glycans and its flexible hinge and tailpiece regions. Thus, protein crystallography has not been suitable for full-length dIgA1. Interestingly, a cryo-electron microscopy structure of the Fc core of secretory polymeric IgA showed that a centrally located J chain connected two Fc regions (21, 22, 23), but this structure showed no information on the positioning of its Fab regions. Instead, small-angle X-ray scattering (SAXS) modeling (24, 25) based on coarse-grained models of the antibody Fab and Fc regions resulted in a near-planar solution structure for human myeloma dIgA1 (26). The advent of atomistic scattering modeling and improved data collection methods resulted in an asymmetric extended solution structure for monomeric human IgA1 (27). Accordingly, the comparison of a multidisciplinary study of scattering-modeled dIgA1 solution structures from IgAN patients with their observed phenotypes will update our modeling of dIgA1 and may provide molecular insights into the pathogenesis of IgAN. In this study, we have analyzed structures for full-length human dIgA1 purified from four subjects and compared the SAXS atomistic modeling results from these with the propensity for (i) mesangial IgA deposition; (ii) ability to generate renal injury both *in vitro* and *in vivo*, and (iii) the pattern of IgA1 *N*-linked and *O*-linked glycosylation. We also showed that the higher *O*-glycosylation of the hinge region was correlated with larger radii of gyration (R_g_) values from the SAXS data, and that the atomistic modeling of the four dIgA1 structures showed that the three dIgA1 samples associated with IgAN showed a slightly more compact structure between the two monomers compared to the healthy control dIgA1. These results provide new insights on the relationship of the dIgA1 composition and structure to the onset of IgAN.

## Results

### Dimeric IgA1 O-linked and N-linked glycosylation

**Figure 2:**
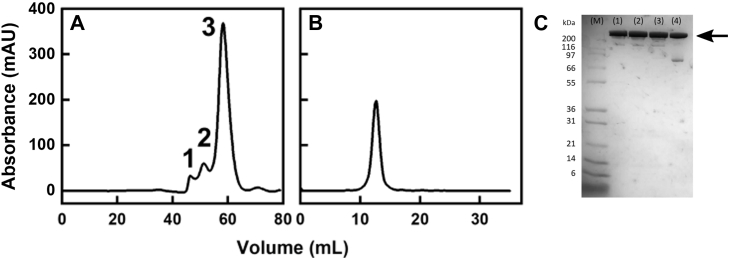
**Purification of dimeric IgA1 by fast performance liquid chromatography of IgA1**. *A*, IgA1 purified from serum was separated into (1) high molecular weight immune complexes (pIgA1), (2) dimer [dimeric IgA1 (dIgA1)], and (3) monomer (mIgA1), using a Superdex 200 pg column under the control of an ÄKTA Prime system. *B*, the dimeric IgA1 fractions were collected, pooled, and concentrated before being passed through a Superose 6 column to further purify the dIgA1 by removing nonspecific aggregates. *C*, nonreducing SDS-PAGE of dIgA1 from the four subjects, namely healthy control and patients *A*, *B*, and *C* in that order (Lanes 1–4). IgA1, immunoglobulin A1.

**Figure 3:**
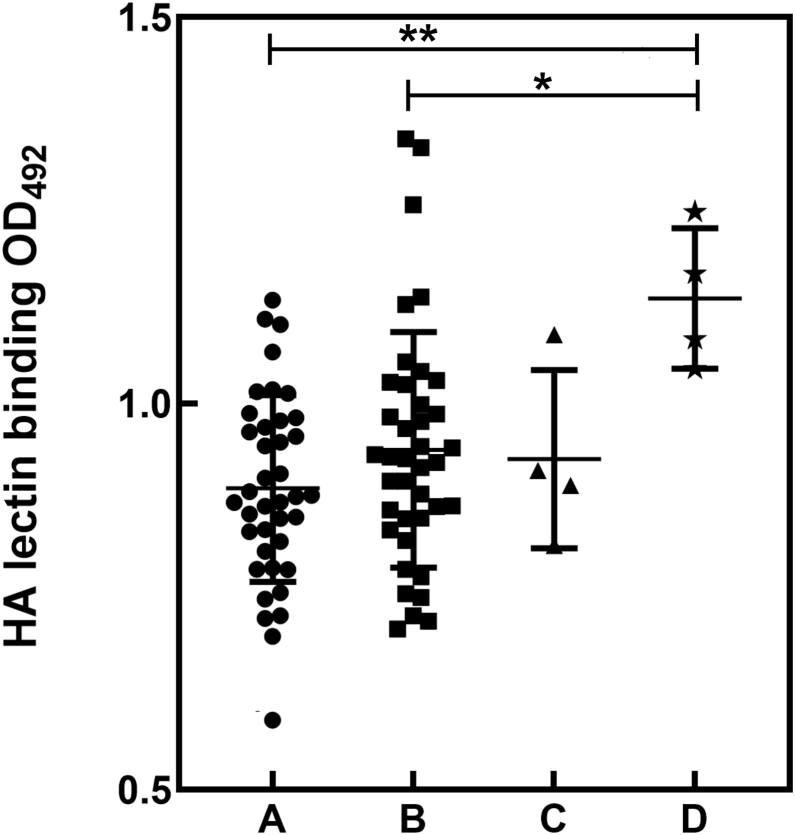
**HAA lectin binding of serum and dimeric IgA1 from healthy subjects and patients with IgAN**. *A–D*, HAA lectin binding of total serum IgA1 from 40 healthy subjects (*A*) and 40 patients with IgAN (*B*) was compared with that of four dIgA1 from healthy subjects (*C*) and four dIgA1 from IgAN patients (*D*). Dimeric IgA1 HAA lectin binding was significantly higher in IgAN (*D*) compared to that seen in total serum IgA1 from healthy subjects (*A*) (*p* < 0.01, marked with double asterisk ∗∗) and total IgA1 from IgAN patients (*B*) (*p* < 0.05, marked with a single asterisk ∗). dIgA1, dimeric IgA1; HAA, *Helix aspersa* agglutinin; IgA, immunoglobulin A; IgAN, IgA nephropathy.

Dimeric IgA was cleanly purified as a single peak from all four subjects (Table 1 and Fig. 2) and characterized as follows (28). As expected, the number of galactose-deficient *N*-acetlygalactosamine residues, as measured by *Helix aspersa* agglutinin (HAA) lectin binding, varied between healthy individuals and IgAN patients and was increased in the dIgA1 fractions compared to total serum IgA1 from the same individual (Fig. 3). There were, however, no major differences in the proportion of the different hinge region *O*-glycoforms between the four subjects measured by mass spectrometry (Table 2, Table 3, Table 4). Similarly, there were no major differences in *N*-glycopeptide distribution in dIgA1 between the four subjects (Table 5). The mean number of *O*-glycans per hinge region glycopeptide and proportion of *N*-glycans per α heavy chain is shown in Table 2, and these were used to inform the dIgA1 structural modeling below. The observed disparity between differences in HAA lectin binding (Fig. 3) and the LC-MS data (Table 2, Table 3, Table 4, Table 5) supports the hypothesis that it is not the absolute number of sugar moieties at the hinge region that determines the pathogenicity of dIgA1 but rather the position of the *O*-glycan attachment within the hinge region (29).

**Table 1:** Baseline characteristics of the four study subjects

	Healthy control	Patient A	Patient B	Patient C
Age (years)	50	65	24	55
Sex	Female	Male	Female	Male
eGFR^a^ (ml/min per 1.73 m^2^)	-	80	75	30
Proteinuria (mg/mmol)	-	35	69.5	120
Clinical course^b^	-	Non-progressor	Progressor	Progressor
Oxford score	-	M0 E0 S0 T0 C0	M1 E1 S0 T0 C1	M1 E0 S1 T1 C0

a eGFR: Estimated glomerular filtration rate.

b A nonprogressor is defined as an estimated eGFR >60 ml/min per 1.73 m^2^ and less than 20% decrease over at least 5 years of follow-up. A progressor is defined as the doubling of serum creatinine or needing kidney replacement therapy during follow-up.

**Table 2:** IgA1 and J chain glycopeptides measured by LC-MS mass spectrometry

Peptide sequence	Glycan linkage	Acronym^a^
IgA1 (UniProtP01876)		
HYTNPSQDVTVPCPVPS^106^TPP^109^TP^111^SP^113^S^114^TPP^117^TPSPSCCHPR	*O*-linked	HYT
LSLHRPALEDLLLGSEA^144^NLTCTLTGLR	*N*-linked	LSL
LAGKPTHV^340^NVSVVMAEVDGTCY	*N*-linked	LAGY
LAGKPTHV^340^NVSVVMAEVDGTC (previously observed truncation)	*N*-linked	LAGC
J-chain (UniProt P01591)		
E^71^NISDPTSPLR	*N*-linked	ENI
IIVPLNNRE^71^NISDPTSPLR (missed cleavage for ‘ENI’ peptide)	*N*-linked	IIV

a The three-letter or four-letter acronyms are used as glycopeptide identifiers.

**Table 3:** The mean number of *O*-glycans per hinge region glycopeptide and proportion of *N*-glycans per α heavy chain and J chain^a^

	HYT (IgA1 *O*-glycopeptide)^b^				
Sample	#H	#S	#N	S/H	H/N
Healthy control	3.86	2.65	4.55	0.68	0.85
Patient A	3.85	2.39	4.52	0.62	0.85
Patient B	3.79	2.84	4.60	0.75	0.82
Patient C	3.75	2.22	4.50	0.59	0.83


Sample	LAGC (IgA1 Asn340)^c^					LAGY (IgA1 Asn340)						
	Total Diantennary *N*-glycans	Total Triantennary *N*-glycans	Bissecting Diantennary *N*-glycans	Sialylated Diantennary *N*-glycans	Sialylated Triiantennary *N*-glycans	Total Diantennary *N*-glycans	Total Triantennary *N*-glycans	Galactosylated Diantennary *N*-glycans	Bissecting Diantennary *N*-glycans	Fucosylated Diantennary *N*-glycans	Sialylated Diantennary *N*-glycans	Sialylated Triiantennary *N*-glycans
Healthy control	100.00%		66.67%	92.60%		97.99%	2.01%	99.53%	45.98%	65.39%	81.75%	47.71%
Patient A	89.80%	10.20%	51.43%	92.52%	61.10%	97.57%	2.43%	98.70%	48.39%	64.86%	71.53%	44.61%
Patient B	100.00%		58.53%	94.87%		99.07%	0.93%	100.00%	49.00%	67.35%	86.28%	33.33%
Patient C	96.69%	3.31%	47.83%	93.75%	55.26%	98.69%	1.31%	98.17%	42.07%	63.94%	72.90%	33.33%


Sample	ENI (J-chain Asn71)							IIV (J-chain Asn71)						
	Total Diantennary *N*-glycans	Total Hybrid type *N*-glycans	Galactosylated Diantennary *N*-glycans	Bissecting Diantennary *N*-glycans	Fucosylated Diantennary *N*-glycans	Sialylated Diantennary *N*-glycans	Fucosylated Hybrid type *N*-glycans	Total Diantennary *N*-glycans	Total Hybrid type *N*-glycans	Galactosylated Diantennary *N*-glycans	Bissecting Diantennary *N*-glycans	Fucosylated Diantennary *N*-glycans	Sialylated Diantennary *N*-glycans	Fucosylated Hybrid type *N*-glycans
Healthy control	84.99%	15.01%	99.71%	3.12%	18.65%	80.37%	82.96%	69.97%	30.03%	100.00%	0.00%	86.18%	89.75%	100.00%
Patient A	81.16%	18.84%	99.28%	4.28%	24.61%	80.30%	75.13%	67.44%	32.56%	99.95%	0.10%	90.89%	89.35%	99.71%
Patient B	88.08%	11.92%	99.56%	2.60%	17.10%	80.41%	85.65%	74.64%	25.36%	100.00%	1.25%	77.07%	87.37%	100.00%
Patient C	75.87%	24.13%	99.22%	3.02%	39.53%	76.10%	87.53%	71.71%	28.29%	100.00%	0.00%	100.00%	88.48%	100.00%

a Each glycopeptide is represented by the first three letters in each sequence (see Table 2 for peptide sequence).

b For the hinge region *O*-glycopeptide, #H is the number of galactoses, #S is the number of sialic acids per glycopeptide, and #N is the number of *N*-acetylgalactosamines.

c In the following text, the four *N*-glycopeptides are abbreviated: Hx [where x is the number of hexoses (galactose or mannose)]; Ny [where y is the number of N-acetylhexosamines (GlcNAc or GalNAc)]; Fz (where z is the number of fucoses), and Sn (where n is the number of sialic acids). *N*-glycans with H_3−5_N_4−5_F_0−1_S_0−2_ were considered to be diantennary, for which the presence of 4 or 5 hexoses is counted as 50 or 100% galactosylation, *N*-acetylhexosamines were counted as bisection, one or two sialic acids were counted as 50 or 100% sialylation. H_5-6_N_3_ and H_6_N_4_ species were considered as hybrid type glycans, and H_6_N_5_ species as triantennary. The total level of these glycans was calculated, as well as the levels of sialylation, bisection, fucosylation, galactosylation, and sialic acid per galactose, if applicable.

**Table 4:** The distribution of hinge region *O*-glycopeptides in dimeric IgA1 from four subjects

	HYT-H2N3S2	HYT-H2N3S3	HYT-H3N3S1	HYT-H3N3S2	HYT-H3N3S3	HYT-H3N3S4	HYT-H2N4S2	HYT-H3N4S0
Healthy control	0.35%		1.38%	2.18%	1.22%	0.16%	0.29%	0.19%
Patient A	0.49%	0.02%	1.06%	2.20%	2.19%	0.43%	0.27%	1.15%
Patient B	0.04%		0.33%	1.48%	1.41%		0.33%	
Patient C	0.71%	0.03%	1.76%	2.63%	1.79%	0.38%	0.35%	1.93%


	HYT-H3N4S1	HYT-H3N4S2	HYT-H3N4S3	HYT-H3N4S4	HYT-H4N4S0	HYT-H4N4S1	HYT-H4N4S2	HYT-H4N4S3
Healthy control	2.81%	3.70%	2.55%	0.42%	0.07%	2.80%	7.55%	10.74%
Patient A	2.75%	2.72%	2.38%	0.35%	1.97%	5.15%	6.57%	9.72%
Patient B	1.09%	4.41%	4.98%	0.64%	0.01%	0.94%	5.36%	10.56%
Patient C	3.36%	3.78%	2.53%	0.35%	2.31%	5.29%	5.98%	8.49%


	HYT-H4N4S4	HYT-H2N5S1	HYT-H2N5S2	HYT-H3N5S0	HYT-H3N5S1	HYT-H3N5S2	HYT-H3N5S3	HYT-H4N5S0
Healthy control	8.08%	0.30%	0.21%	0.04%	1.39%	2.66%	2.88%	0.14%
Patient A	7.92%	0.24%	0.23%	0.96%	1.72%	2.13%	2.34%	1.50%
Patient B	9.82%	0.10%	0.79%	0.01%	1.08%	4.55%	6.19%	0.01%
Patient C	6.22%	0.45%	0.32%	1.33%	2.74%	2.60%	2.54%	1.78%


	HYT-H4N5S1	HYT-H4N5S2	HYT-H4N5S3	HYT-H4N5S4	HYT-H4N5S5	HYT-H5N5S0	HYT-H5N5S1	HYT-H5N5S2
Healthy control	3.33%	8.32%	12.59%	8.52%	0.21%	0.04%	0.83%	2.37%
Patient A	4.77%	6.55%	9.19%	7.22%	0.14%	0.59%	1.85%	2.15%
Patient B	1.40%	7.34%	15.42%	9.07%	0.09%	0.00%	0.47%	1.68%
Patient C	5.23%	6.20%	10.13%	5.53%	0.10%	0.47%	1.34%	1.68%


	HYT-H5N5S3	HYT-H5N5S4	HYT-H5N5S5	HYT-H3N6S0	HYT-H3N6S1	HYT-H3N6S2	HYT-H4N6S0	HYT-H4N6S2
Healthy control	4.02%	2.79%	0.14%	0.02%	0.25%	0.61%	0.02%	0.75%
Patient A	3.46%	2.12%	0.12%	0.14%	0.48%	0.73%	0.16%	0.66%
Patient B	3.36%	2.02%		0.01%	0.24%	0.91%	0.00%	0.77%
Patient C	2.67%	1.40%	0.08%	0.25%	0.82%	0.92%	0.23%	0.69%


	HYT-H4N6S3	HYT-H4N6S4	HYT-H5N6S0	HYT-H5N6S3	HYT-H5N6S4
Healthy control	1.65%	0.41%		0.75%	0.28%
Patient A	1.62%	0.45%	0.08%	0.70%	0.36%
Patient B	1.52%	0.59%		0.60%	0.34%
Patient C	1.45%	0.32%	0.06%	0.55%	0.21%

The hinge glycopeptide is represented by HYT (see Table 2 for peptide sequence).

Hx: the number of galactoses; Nx: the number of *N*-acetylgalactosamines, and Sx: the number of sialic acids per glycopeptide.

**Table 5:** The distribution of *N-*glycopeptides in the heavy chain of dIgA1 and its J chain from four subjects

dIgA1	LAGC-H5N4F1S1	LAGC-H5N4F1S2	LAGC-H5N5F1S1	LAGC-H5N5F1S2	LAGC-H6N5F1S1	LAGC-H6N5F1S2	LAGC-H6N5F1S3
Healthy control	3.87%	29.46%	10.94%	55.73%			
Patient A	5.19%	38.43%	8.24%	37.94%	2.00%	7.90%	0.30%
Patient B		41.47%	10.25%	48.28%			
Patient C	5.37%	45.07%	6.72%	39.54%	1.13%	2.18%	


dIgA1	LAGY-H4N5F0S0	LAGY-H4N5F1S0	LAGY-H5N4F0S1	LAGY-H5N4F0S2	LAGY-H5N4F1S1	LAGY-H5N4F1S2	LAGY-H5N5F0S1
Healthy control	0.45%	0.47%	8.10%	20.37%	4.07%	20.40%	4.99%
Patient A	0.26%	2.28%	13.44%	13.79%	6.83%	16.30%	6.79%
Patient B			8.19%	19.80%	4.28%	18.26%	4.35%
Patient C	0.33%	3.28%	15.17%	14.60%	7.93%	19.48%	5.50%


dIgA1	LAGY-H5N5F1S0	LAGY-H5N5F1S1	LAGY-H5N5F1S2	LAGY-H6N5F1S1	LAGY-H6N5F1S2
Healthy control	1.23%	14.30%	23.61%	1.14%	0.87%
Patient A	2.35%	18.71%	16.82%	1.61%	0.82%
Patient B		10.37%	33.82%	0.93%	
Patient C	0.59%	16.51%	15.32%	1.31%	


J chain	ENI-H4N5F0S0	ENI-H5N3F0S1	ENI-H5N3F1S1	ENI-H5N4F0S1	ENI-H5N4F0S2	ENI-H5N4F1S0	ENI-H5N4F1S1
Healthy control	0.49%	2.56%	12.43%	24.82%	41.67%	0.01%	5.38%
Patient A	1.16%	4.68%	14.12%	20.95%	36.77%	0.01%	6.36%
Patient B	0.78%	1.71%	10.21%	25.56%	45.16%	0.06%	5.75%
Patient C	1.18%	3.01%	19.58%	20.11%	23.49%	0.03%	12.64%


J chain	ENI-H5N4F1S2	ENI-H5N5F0S1	ENI-H6N3F1S1
Healthy control	10.45%	2.16%	0.02%
Patient A	13.60%	2.31%	0.03%
Patient B	9.25%	1.51%	
Patient C	17.33%	1.11%	1.54%


J chain	IIV-H4N5F0S0	IIV-H5N3F0S1	IIV-H5N3F1S1	IIV-H5N4F0S1	IIV-H5N4F0S2	IIV-H5N4F1S1	IIV-H5N4F1S2
Healthy control			30.03%	2.27%	7.40%	12.07%	48.23%
Patient A	0.07%	0.09%	32.47%	1.31%	4.76%	12.92%	48.38%
Patient B			25.02%	4.21%	11.96%	13.70%	43.83%
Patient C			28.29%			16.52%	55.19%


J chain	IIV-H5N5F0S1	IIV-H6N3F1S1
Healthy control		
Patient A		
Patient B	0.94%	0.33%
Patient C		

Each glycopeptide is identified by either a three or four letter code (see Table 2 for the peptide sequences).

Fx, the number of fucoses; Hx, the number of hexoses (galactose or mannose); Nx, the number of *N*-acetylhexosamines (GlcNAc or GalNAc); and Sx, the number of sialic acids per glycopeptide.

### IgA–IgG immune complex formation and human mesangial cell activation by dIgA1

**Figure 4:**
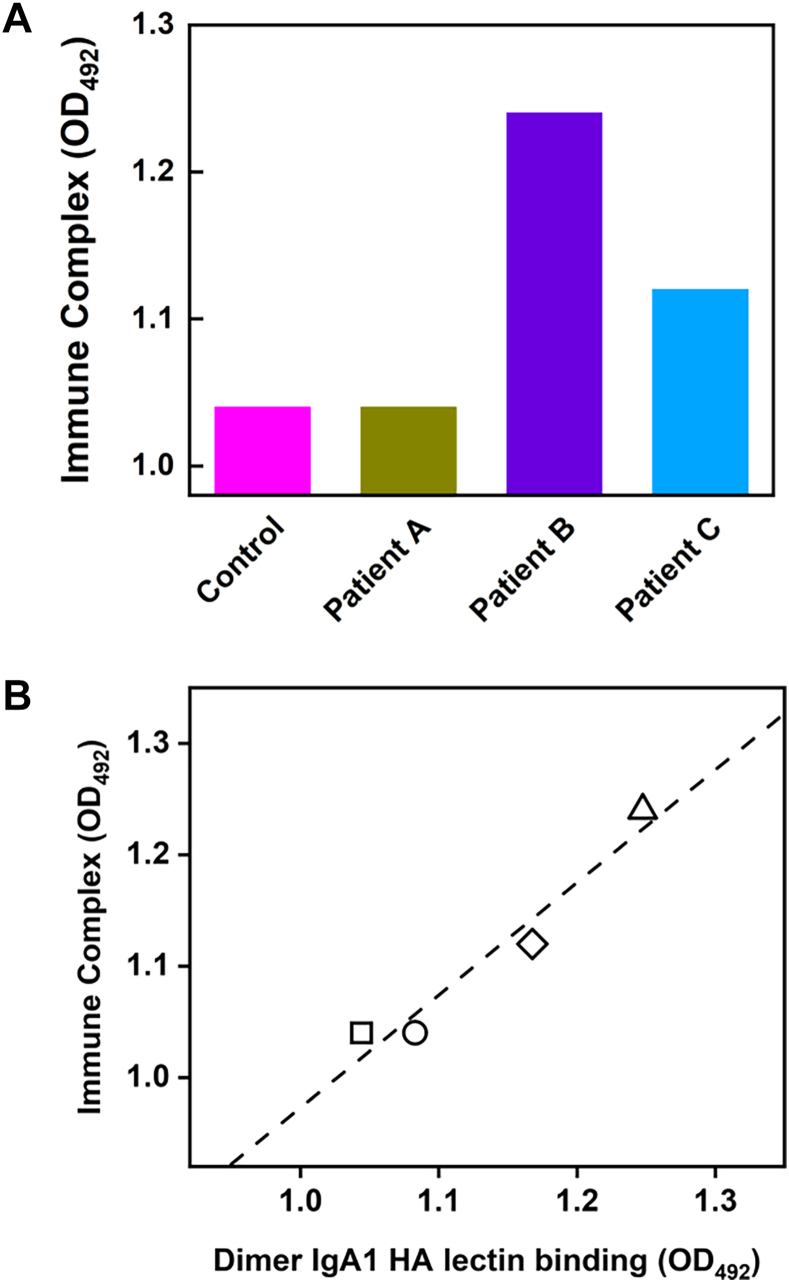
**dIgA1–IgG immune complex levels in the four subjects.***A*, levels of dIgA1–IgG immune complexes in the serum of each of the four experimental subjects were measured using a bespoke ELISA, where the relative levels were displayed as *A*_492_ (healthy control, *pink*; patient A, *olive*; patient B, *purple*; patient C, *blue*). *B*, relationship between the extent of dIgA1 hinge region *O*-galactosylation, as measured using a HA ELISA, and the level of IgA–IgG immune complexes in the same serum sample in the four experimental subjects (healthy control, ◯; patient A, □; patient B, △; patient C, ◇). dIgA1, dimeric IgA1.

Assays show that the levels of IgA–IgG immune complexes formed in serum varied in the four subjects (Fig. 4*A*). While we cannot be certain that these immune complexes are exclusively formed between poorly *O*-galactosylated IgA1 and IgG antiglycan antibodies, the levels of these complexes correlated linearly with the HAA assay for the lectin binding of dIgA1, consistent with the hypothesis that changes in hinge region *O*-galactosylation promote IgA–IgG immune complex formation (Fig. 4*B*).

**Figure 5:**
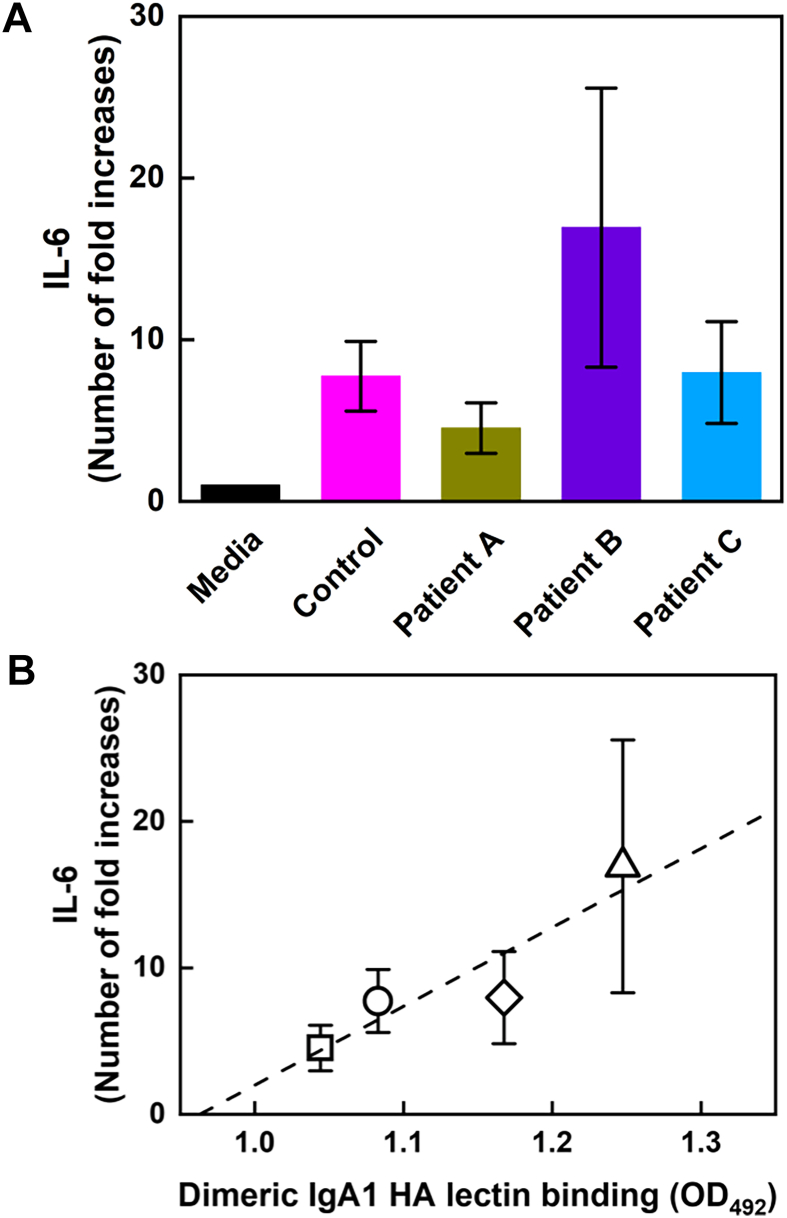
**Dimeric IgA1-induced IL-6 synthesis in human mesangial cells (hMC)**. *A*, human mesangial cells were exposed to dIgA1 (50 μg/ml) from each of the four subjects for 48 h. IL-6 concentration was measured in the tissue culture supernatant by ELISA and is displayed as fold increase compared to media only (mean ± SEM, n = 4). There was a variable increase in secreted IL-6 in response to exposure to dIgA1 (healthy control, *pink*; patient A, *olive*; patient B, *purple*; patient C, *blue*). *B*, relationship between the extent of dIgA1 hinge region *O*-galactosylation, as measured using a HA ELISA, and the dimeric IgA1-induced IL-6 response in human mesangial cells in the four experimental subjects (healthy control, ◯; patient A, □; patient B, △; patient C, ◇). dIgA1, dimeric IgA1.

Dimeric IgA1 from each subject stimulated human mesangial cell (hMC) synthesis of interleukin-6 (IL-6) although the magnitude of the IL-6 response varied considerably (Fig. 5*A*). The IL-6 response also correlated closely with the HAA lectin binding of dIgA1, consistent with the hypothesis that changes in hinge region *O*-galactosylation promote glomerular inflammation and renal injury in IgAN (Fig. 5*B*).

Consistent with these *in vitro* observations, patient A, who had the lowest level of HAA lectin binding, lowest level of IgA–IgG immune complexes in the serum, and smallest IL-6 response, had the least active renal biopsy Oxford score (M0, E0, S0, T0, C0) and showed the best long-term renal survival, best kidney function, and lowest proteinuria (28). By contrast, patient B, who had the highest level of HAA lectin binding, the highest level of IgA–IgG immune complexes in the serum, and the greatest IL-6 response, had the most active renal biopsy Oxford score (M1, E1, S0, T0, C1) and progressive IgAN.

### Analytical ultracentrifugation of dIgA1

**Figure 6:**
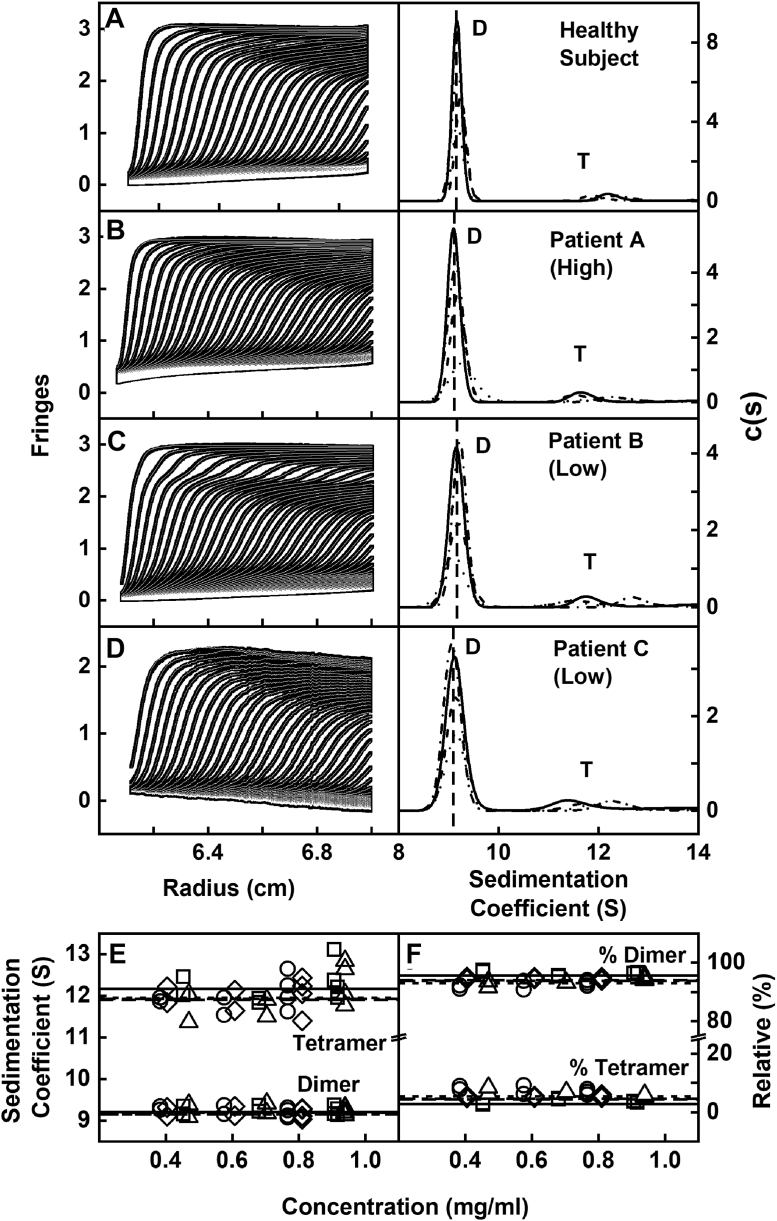
**Sedimentation velocity analyses of dIgA1**. *A–D*, the experimentally observed sedimentation boundaries for dIgA1 samples from the healthy subject and patients *A*, *B*, and *C* in PBS buffer were recorded at rotor speeds between 30,000 and 40,000 rpm and 20 °C. Approximately 30 boundaries (*black outlines*) are shown for up to 150 scans at intervals of about every fifth scan for clarity, fitted using SEDFIT as shown (*thin white lines*). The *right-hand panel* shows the size-distribution analyses *c*(*s*), revealing a dimer (*D*) peak at *s*_*20,w*_ values of 9.2 to 9.3 S for the four dIgA1 samples and shown in triplicate. Small amounts of a tetramer (T) were detectable at about 12 S. *E*, the *s*_*20,w*_ values for dimeric and tetrameric IgA1 are shown as a function of dIgA1 concentration (healthy subject, ◯; patient A, □; patient B, △; patient C, ◇). *F*, the percentages of dimer and tetramer in each dIgA1 sample from integration of the *c(s)* analyses are shown. dIgA1, dimeric IgA1.

Analytical ultracentrifugation (AUC) uses macromolecular sedimentation under a high centrifugal force to determine masses and solution structures (30). This was used to compare the four dIgA1 samples. The SEDFIT analyses of 32 separate dIgA1 runs in light water phosphate-buffered saline (PBS) buffer in concentration ranges of 0.38 to 0.94 mg/ml and eight separate dIgA1 runs in heavy water involved fits of as many as 150 scans. These showed good agreement between the experimental boundary scans and fitted lines (left panels; Fig. 6, *A*–*D*). The resulting size distribution analyses *c(s)* showed that all four dIgA1 samples were predominantly dimeric in solution (peak D), and all were accompanied by a minor tetrameric peak (peak T; approximately 3–8%) (Fig. 6, *E* and *F*). The dimer peak was observed at *s*^*0*^_*20,w*_ values of 9.2 to 9.3 S for all dIgA1 samples (right panels; Fig. 6, *A*–*D*), consistent with previously reported values (26). For 0.91 mg/ml healthy control dIgA1, the *s*^*0*^_*20,w*_ values at 30,000 rpm and 40,000 rpm were similar (9.4 S and 9.3 S respectively), thus almost no dependence on rotor speed was seen that would otherwise imply the presence of very flexible hinge regions. All the dIgA1 data reported in this study were for 40,000 rpm. The mean *s*^*0*^_*20,w*_ values of the dIgA1 were 9.25 ± 0.09 S (healthy control), 9.23 ± 0.10 S (patient A), 9.25 ± 0.11 S (patient B), and 9.20 ± 0.11 S (patient C), and the dIgA1 sedimentation rates did not depend on the concentration (Table 1). The *c(s)* analyses indicated that the average molecular masses of the dIgA1 peaks were 319 ± 6 kDa (healthy control), 316 ± 4 kDa (patient A), 317 ± 5 kDa (patient B), and 314 ± 4 kDa (patient C) in PBS in H_2_O. These agreed well with the expected molecular mass of dIgA1 of ∼320 kDa and supported the identification of the peak as the IgA1 dimer.

### SAXS analyses of dIgA1

The solution structures of the four dIgA1 samples were characterized by SAXS and small-angle neutron scattering (SANS). The two methods differ in that X-rays in H_2_O buffer detect the hydration shell surrounding dIgA1, while neutrons in D_2_O buffers reveal a much-reduced view of the hydration shell because of the different solute-solvent contrast in use (24, 25). The two data collections thus provide tests of self-consistency of each other.

In total, 48 separate runs were measured by SAXS for the dIgA1 samples from four subjects (comprising of four different concentrations with triplicates at each concentration for each subject). It was noted that runs with less than four frames exposure may indicate that radiation damage or X-ray induced aggregation effects were present at longer exposures, and hence these later runs were excluded from analysis. As such, seven time-averaged runs were excluded, and a total of 41 time-averaged runs were included in the analysis (healthy control, 11 runs; patient A, 10 runs; patient B, 12 runs; patient C, eight runs).

**Figure 7:**
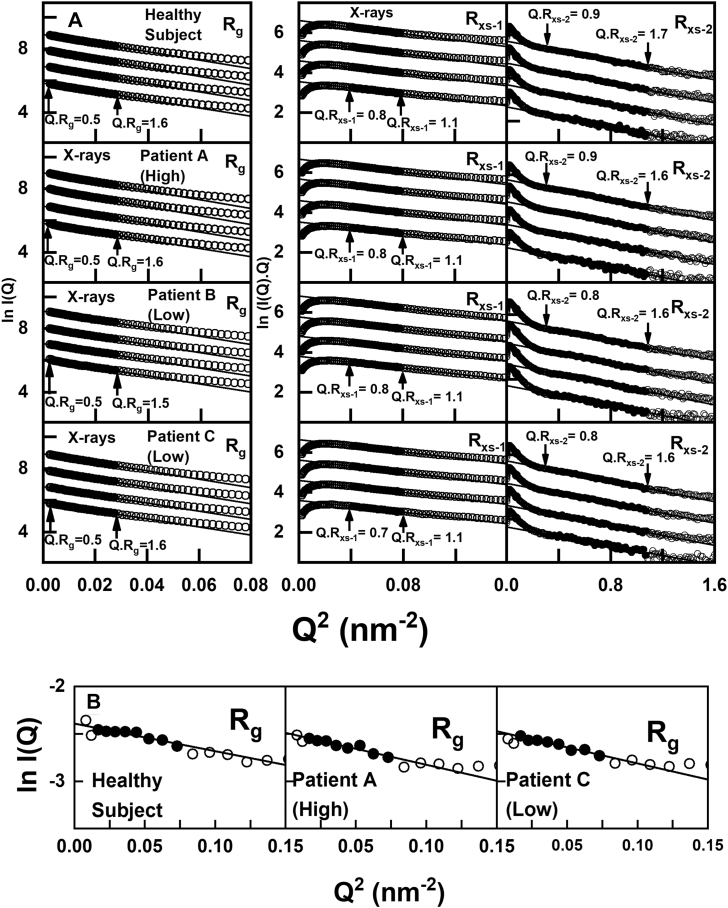
**X-ray and neutron Guinier *R*_*g*_ and *R*_*xs*_ analyses for dIgA1.***A*, the X-ray scattering curves from bottom to top for 0.33, 0.66, 0.98, and 1.31 mg/ml of dIgA1 from the healthy subject; 0.31, 0.62, 0.92, and 1.23 mg/ml of dIgA1 from patient A; 0.32, 0.63, 0.95, and 1.26 mg/ml of dIgA1 from patient B; and 0.28, 0.57, 0.85, and 1.13 mg/ml of dIgA1 from patient C, all measured at 20 °C in PBS-137. The *filled circles* between the arrowed data points represent the *Q*.*R*_*g*_ and *Q*.*R*_*xs*_ ranges used to determine the *R*_*g*_ and *R*_*xs*_ values. The *Q*-ranges for the *R*_*g*_ values was 0.06 to 0.17 nm^−1^. The *Q*-ranges for the *R*_*xs−1*_ and *R*_*xs−2*_ values were 0.20 to 0.28 nm^−1^ and 0.56 to 1.04 nm^−1^, respectively. *B*, the neutron scattering curves for 0.95 mg/ml of dIgA1 from the healthy subject; 0.71 mg/ml of dIgA1 from patient A; and 0.84 mg/ml of IgA1 from patient C are shown for PBS-137 buffer in ^2^H_2_O at 20 °C. The *Q*-ranges for the *R*_*g*_ values was 0.06 to 0.28 nm^−1^. The *Q*-ranges for the *R*_*xs−1*_ and *R*_*xs−2*_ values were 0.20 to 0.28 nm^−1^ and 0.56 to 1.04 nm^−1^, respectively (data not shown). dIgA1, dimeric IgA1; R_g_, radius of gyration.

The Guinier radius of gyration (*R*_*g*_) value monitors the overall shape of dIgA1. At the lowest *Q* value range of 0.06 to 0.17 nm^−1^, the Guinier analyses of ln *I*(*Q*) against *Q*^2^ resulted in linear plots for all dIgA1 samples. These *R*_*g*_ values fell within satisfactory $Q\cdot R_{g}$ limits in the plots and hence yielded the *R*_*g*_ value from their slopes (Fig. 7*A*). The selected *Q* range for the *R*_*g*_ analysis was similar to the range previously determined of 8.65 ± 0.27 nm (26). The mean *R*_*g*_ values for the healthy control and the IgAN patients A, B, and C were determined as 8.80 ± 0.11 nm (11 values), 8.74 ± 0.13 nm (10 values), 8.56 ± 0.09 nm (12 values), and 8.61 ± 0.14 nm (8 values) respectively.

**Figure 8:**
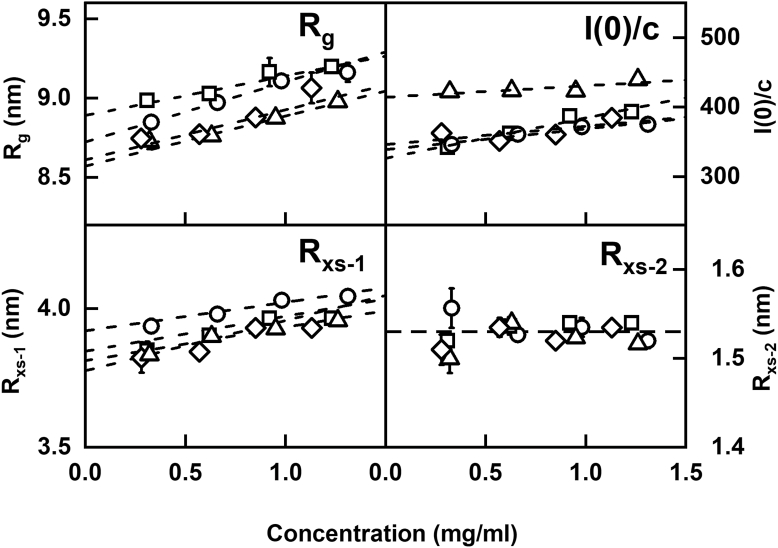
**Concentration dependence of the Guinier values for dIgA1.** The X-ray *R*_*g*_, *I*(*0*)/*c*, *R*_*xs−1*_ and *R*_*xs−2*_ values for the four dIgA1 samples were each measured in triplicate and averaged to give the mean ± standard deviation. Error bars are shown only when visible. The values correspond to the healthy subject (◯), patient A (□), patient B (△), and patient C (◇). The *R*_*g*_, *R*_*xs−1*_ and *I*(*0*)/*c* data were fitted by linear regression, whereas the *R*_*xs−2*_ data were fitted using their mean value. dIgA1, dimeric IgA1; R_g_, radius of gyration.

In addition, the *R*_*g*_ values also increased with concentration for all four subjects (Fig. 8), this being attributed to the observation of small tetramer formation with increase of concentration seen in the AUC analyses (Fig. 6). The mean *R*_*g*_ (± standard error of mean) was thus calculated at each concentration and plotted against the concentration to determine the correlation between *R*_*g*_ and concentration (Fig. 8). The best fit $R_{g}^{0}$ (defined as *R*_*g*_ when this was extrapolated to 0 mg/ml) was calculated by regression analysis. The regression analysis demonstrated a good fit, with *r*^2^ values between 0.58 and 0.80 and no statistically significant differences between the slopes for all subjects. The calculated $R_{g}^{0}$ for dIgA1 was 8.75 ± 0.05 nm, 8.90 ± 0.06 nm, 8.62 ± 0.05 nm, and 8.62 ± 0.07 nm for the healthy control and patients A, B, and C respectively. It was of interest that the $R_{g}^{0}$ for subjects with high hinge *O*-galactosylation (healthy control and patient A) were significantly higher than subjects with low *O*-galactosylation (patients B and C).

The *R*_*xs*−1_ and *R*_*xs*−2_ parameters monitor the two shorter axes of the dlgA1 shape. Using larger *Q* values, analyses of ln *I*(*Q*).*Q* against *Q*^2^ yielded the *R*_*g*_ value of the cross-section (*R*_*xs*−1_ and *R*_*xs*−2_). The *Q* ranges for *R*_*xs*−1_ and *R*_*xs*−2_ were selected, where the ln *I*(*Q*).*Q* against *Q*^2^ showed two linear regions, and the ranges selected were similar to those described previously in the study of dIgA1 (0.20–0.28 nm^−1^ for *R*_*xs*−1_ and 0.56–1.04 nm^−1^ for *R*_*xs*−2_) (26). The mean *R*_*xs*−1_ for the control and patients A, B, and C were 3.99 ± 0.05 nm, 3.91 ± 0.06 nm, 3.90 ± 0.05 nm, and 3.87 ± 0.08 nm, respectively (Fig. 7*A*). Similar to the *R*_*g*_ values, the *R*_*xs*−1_ values also increased with concentration over the concentration series for all subjects (Fig. 8). The mean *R*_*xs*−1_ was thus calculated at each concentration to determine the correlation, and the best fit $R_{xs-1}^{0}$ (defined as the *R*_*xs*−1_ value at 0 mg/ml) was calculated by regression analysis (Fig. 8). The calculated $R_{xs-1}^{0}$ was 3.90 ± 0.02 nm, 3.82 ± 0.03 nm, 3.80 ± 0.02 nm, and 3.78 ± 0.05 nm for the healthy control and patients A, B, and C, respectively. Here, the $R_{xs-1}^{0}$ value of the healthy control is higher than that of the IgAN patients, although there was no difference between subjects with high or low *O*-galactosylation.

The mean *R*_*xs*−2_ values for the healthy control and patients A, B, and C were 1.53 ± 0.03 nm, 1.53 ± 0.01 nm, 1.52 ± 0.02 nm, and 1.52 ± 0.02 nm, respectively. In contrast to the *R*_*g*_ and *R*_*xs*−1_ values, no correlation was observed between *R*_*xs*−2_ and increasing concentration for all subjects. In addition, there was no difference in the mean *R*_*xs*−*2*_ values between the healthy control and IgAN patients and between high or low *O*-galactosylation. This similarity of the *R*_*xs*−2_ between all subjects and at different concentration and also with previously published results (26) indicated that the Fab regions retain their distinct conformational identity within the dimeric structure. It is also notable that the *R*_*xs*−2_ for dIgA1 is close to that of monomeric IgA1 (27), indicating that the structure of the Fab region is preserved within both the monomeric and dimeric structures.

**Figure 9:**
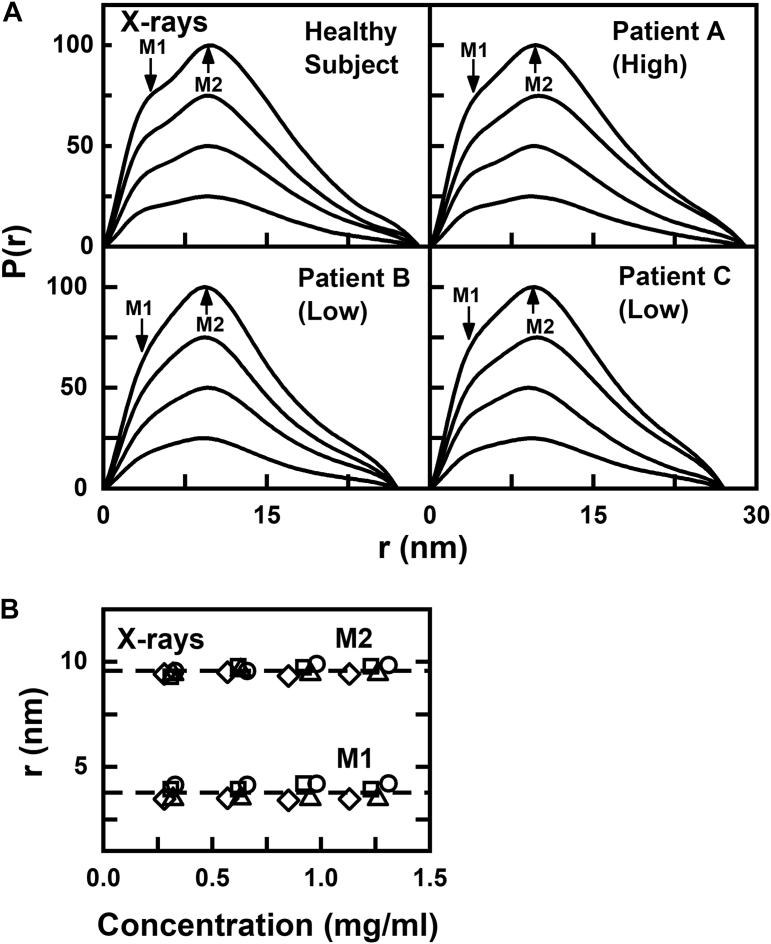
**X-ray distance distribution analyses *P*(*r*) for dIgA1.** In the *P*(*r*) analyses, the peak maxima at *M1* and *M2* are *arrowed*. *A*, the X-ray *P*(*r*) curves for the four dIgA1 samples are shown for concentrations between 0.28 and 1.31 mg/ml, based on the same curves shown in Figure. 7. *B*, the concentration dependences of the maxima *M1* and *M2* in the X-ray *P*(*r*) curves of *A* are shown. The symbols for the healthy subject and three patient samples follow those in Figure. 8. In all cases, the lines represent the mean values. dIgA1, dimeric IgA1.

Transformation of the *I*(*Q*) curve into the distance distribution function *P*(*r*) curve provided structural information on full-length dIgA1 in real space, this being equivalent to a histogram of all the distances between the atoms in dIgA1. The *R*_*g*_ values from the X-ray *P*(*r*) analyses (result not shown) were similar to those from the X-ray Guinier analyses, showing that the two analyses were self-consistent. The maximum length *L* of dIgA1 was determined from the value of *r* when the *P*(*r*) curve intersects zero at large *r* (Fig. 9). The L values were higher at 29.3 ± 0.5 nm for the healthy control and 28.8 ± 0.5 nm for patient A and were reduced at 26.8 ± 0.5 nm for patient B and 27.0 ± 0.8 nm for patient C. The L values increased to 30 nm with increasing concentration for dIgA1 from the healthy subject and the three patients, this being consistent with weak tetramer formation (Fig. 6). The two maxima *M1* and *M2* in the *P*(*r*) curves correspond to the most frequently occurring interatomic distances within the structure. The *M1* peak corresponded mostly to distances that arose within each of the three Fab and Fc regions, whereas the *M2* peak corresponded mostly to distances between the Fab–Fab and Fab–Fc pairs. These two peaks were identified at 5.3 ± 0.1 nm for the healthy control, 5.6 ± 0.2 nm for patient A, 5.9 ± 0.1 nm for patient B, and 5.6 ± 0.3 nm for patient C for *M1* and 9.7 ± 0.2 nm for the healthy control, 9.5 ± 0.1 nm for patient A, 9.2 ± 0.1 nm for patient B, and 9.4 ± 0.1 nm for patient C for *M2* (Fig. 9). No concentration dependence in the positions of peaks *M1* and *M2* was observed, and the four IgA1 samples showed similar *M1* and *M2* values. The current X-ray data resembled those for dIgA1 previously reported.

### SANS analyses of dIgA1

Complementary neutron scattering data on instrument LOQ were obtained for dIgA1. Three single-time frame exposures were obtained for each of the four samples, and the average of the three time frames for each sample was used for analysis. However, compared to the healthy control and patients A and C, the neutron scattering data for patient B revealed a wide-angle distribution plot of ln *I*(*Q*) against *Q* with an abnormal profile with high intensities at low *Q* values at 0 to 0.5 nm^−1^, suggesting the presence of a high molecular weight species such as protein aggregates. Aggregates were subsequently confirmed on FPLC after sample recovery. For this reason, the neutron data from patient B were not analyzed.

The neutron *R*_*g*_ for dIgA1 was 7.27 ± 0.54 nm, 8.62 ± 0.33 nm, and 7.69 ± 0.95 nm for the healthy control, and patients A and C in that order (Fig. 7*B*). The three neutron *R*_*g*_ values supported the X-ray *R*_*g*_ determination, and the good agreement with the X-ray values showed that dIgA1 was stable during X-ray irradiation. In general, the neutron *R*_*g*_ values were less than the X-ray *R*_*g*_ values, and this difference can be attributed to the nonvisibility of the hydration shell by neutrons. The neutron *R*_*xs*−1_ values were calculated to be 4.66 ± 0.20 nm, 4.29 ± 0.42 nm, and 4.14 ± 0.29 nm for the healthy control, and patients A and C respectively, while the neutron *R*_*xs*−2_ were 1.40 ± 0.06 nm, 1.22 ± 0.09 nm, and 1.57 ± 0.07 nm, respectively.

There is a linear relationship between the LOQ Guinier *I*(0)/*c* (c = sample concentration) values for proteins measured in ^2^H_2_O buffers and normalized against a standard deuterated polymer and the protein molecular mass, *M*_*r*_, where *M*_*r*_ = *I*(0)/*c* × 9 × 10^5^. The dIgA1 neutron Guinier *I*(0)/*c* value of 0.35 ± 0.05, 0.43 ± 0.03, and 0.34 ± 0.07 corresponded to *M*_*r*_ values of 315 ± 45 kDa, 387 ± 27 kDa, and 306 ± 63 kDa for the healthy control, and patients A and C respectively. This measurement was within the expected value of 320 kDa for dIgA1, showing that the scattering data corresponded well to the expected size of dIgA1. The neutron *P*(*r*) curves were not analyzed as the X-ray *P*(*r*) curve for dIgA1 was of better quality than the neutron *P*(*r*) for reason of better signal-noise ratios.

### Atomistic modeling of the solution structure for dIgA1

**Figure 10:**
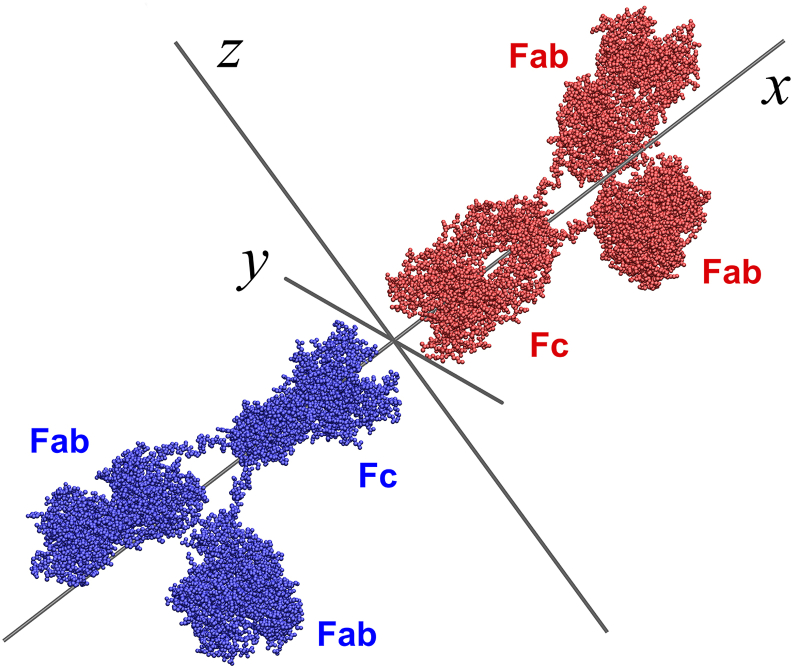
**Dimer rotation modeling.** The Cartesian coordinate system was defined such that the *x*-axis passes across the middle of the Fc region of an IgA1 monomer, shown here in *blue*. The origin was placed a certain distance below the Fc region of the monomer along the *x*-axis. A copy of the monomer, shown in *red*, was created along the *x*-axis and on the other side of the *y*−*z* plane to render the starting dimer model.

Atomistic modeling simulations of the scattering curves for trial dIgA1 structures were performed to locate the two monomeric structures of IgA1 in the dimer structure based on the scattering curves (Fig. 10). The simulations were initiated from the best-fit monomer model for IgA1 from previous study (27). All 75,449 possible geometrical orientations of one monomer relative to the other were computed by 10° rotations for the comparison.

**Figure 11:**
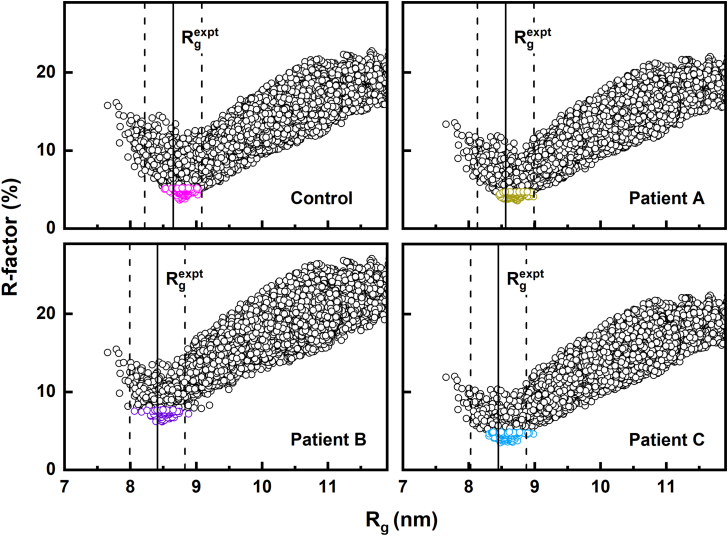
**Atomistic modeling search for the solution structure of dIgA1.** The 28,023 dIgA1 models were fitted to one X-ray experimental scattering curve extrapolated to zero concentration. *Black circles* correspond to all the generated models. The best-fit 100 models with the lowest R-factor values are shown in *magenta*, *dark yellow*, purple, and *light blue* colors for the healthy control, patient A, patient B, and patient C, respectively. The *vertical solid black lines* represent the experimental values of *R*_*g*_, and the *vertical dashed lines* mark the ±5% error margins corresponding to each experimental *R*_*g*_ value. dIgA1, dimeric IgA1; R_g_, radius of gyration.

The simulated scattering curves of the final 28,023 physically plausible, computationally generated atomistic structures were calculated (Experimental Procedures). These were fitted against the experimentally obtained SAXS scattering curves extrapolated to zero concentration of all four samples. To assess the goodness of each fit, an *R*-factor value was calculated. For each simulated structure, an $R_{g}$ value was also calculated using the atomic coordinates within each macromolecular structure. Plots of the *R*-factor against $R_{g}$ for each of the four samples enabled the fits to be summarized (Fig. 11). Lower *R*-factor values indicate better fits. As desired, a clear minimum was seen in each plot, each of which was very close to the experimental $R_{g}$ value for each sample (vertical solid lines). The vertical dashed lines mark the ± 5% error margin on each experimental $R_{g}$ value. On each plot, structures with the lowest 100 *R*-factor values, *i.e.*, the 100 best-fit structures, are shown in colored symbols. Almost all the best-fit, 100 models showed $R_{g}$ values within the ±5% error margin of the experimental $R_{g}$ values, that indicated very good modeling fits.

**Figure 12:**
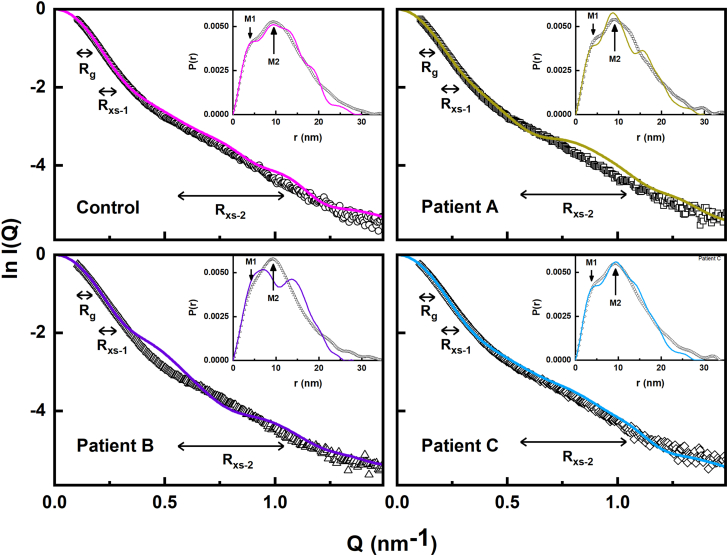
**Scattering curve best fits for dIgA1 models.** The best fits correspond to the models with the lowest R-factor. The experimental data are indicated by symbols (healthy control, ◯; patient A, □; patient B, △; patient C, ◇), overlaid with the modeled scattering curve (*solid line*). The colors of the modeled curves are the same as in Figure. 11. The insets show the distance distribution curves *P*(*r*) found within each of the modeled best-fit structures. dIgA1, dimeric IgA1.

A comparison of the scattering curve of each best-fit structure (corresponding to the minimum in each of the four plots in Fig. 11) showed very good agreements with the corresponding experimental scattering curve (Fig. 12). Also shown are the *Q*-value ranges that were used to calculate the experimental values of $R_{g}$, $R_{xs-1}$ and $R_{xs-2}$. The inset in each frame shows the comparison of the corresponding *P*(*r*) curve, calculated from the scattering curve according to Equation 4, between experiment and the best-fit computational structure. Apart from patient B which showed small deviations close to the main peak, the simulated and experimental *P(r)* curves showed good agreements.

**Figure 13:**
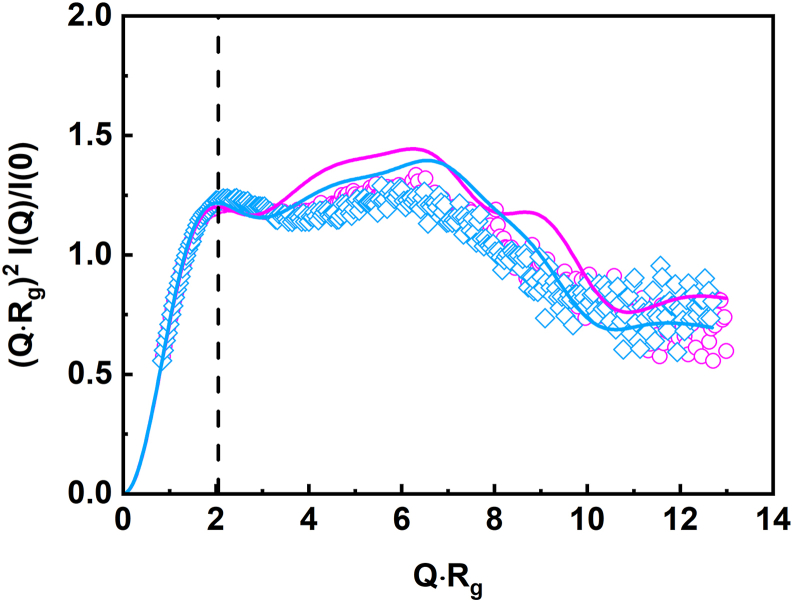
**Normalized dimensionless Kratky plots of the best fit curves of dIgA1 models.** The experimental data for the control sample and patient C are denoted by *magenta circles* and *light blue diamonds*, respectively. The corresponding best-fit modeled curves for the two samples are shown with *solid lines* of the same colors. The vertical *dotted line* shows the peak of the experimental curves, which coincides with the peak of the best fit curves. Rg, radius of gyration.

More structural information was obtained from the Kratky curve, which can identify whether a given structure is extended and flexible, or compact and globular. The normalized, dimensionless Kratky curves for the healthy control and patient C showed a peak at $Q\cdot R_{g}$ values of around 2 (Fig. 13). The experimental curves were reduced at higher $Q\cdot R_{g}$ values. The modeled curves followed the same trends, overall indicating a partially folded, multidomain structure with flexible linkers.

**Figure 14:**
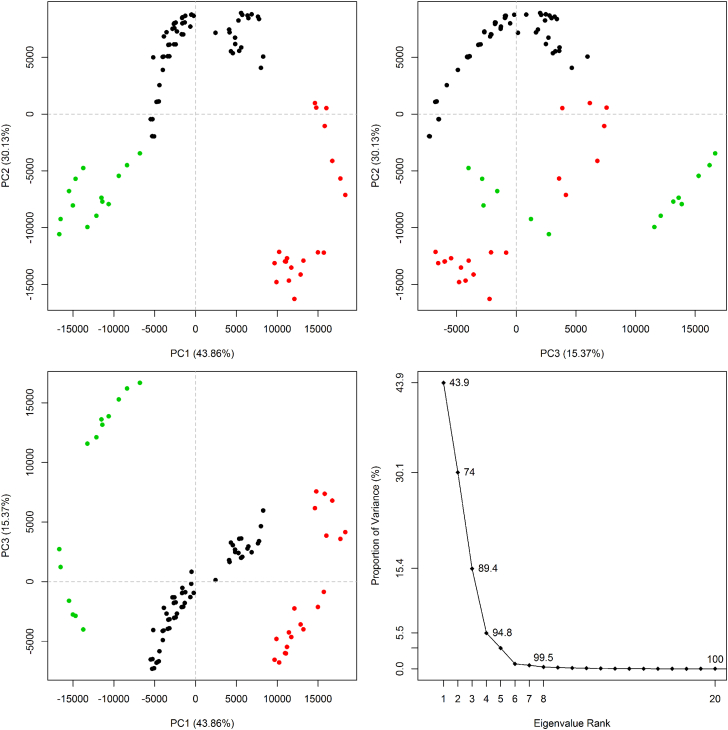
**Principal component analysis of the 100 best-fit models for the healthy control.** The 100 best-fit models were grouped into three principal component groups, denoted with PC1, PC2, and PC3. For the healthy control, these three groups contained 59 (*black*), 21 (*red*), and 20 (*green*) models, respectively. The first three eigenvalue rankings (PC1 to PC3) accounted for 89.4% of the variance.

**Figure 15:**
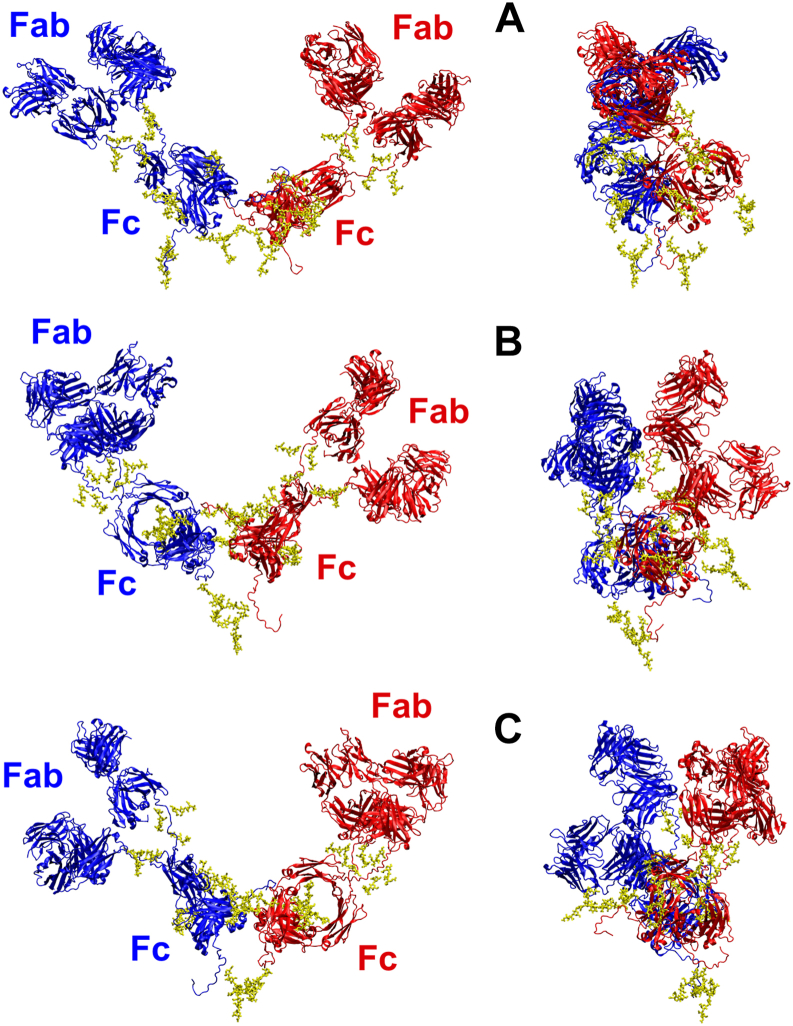
**Representative structures from the three PCA groups for the healthy control.** The *left*-hand side panel shows the front view, whereas the right-hand side panel shows the side-on view. The glycans are shown as *yellow sticks*. The images were produced using the VMD package. *A*, PC1 contained structures in which the Fab arms of the two monomers (*blue* and *red*) face opposite to each other. *B*, PC2 had structures with the Fab arms of the two monomers facing in the same direction (to the *right*). *C*, PC3 had the Fab arms roughly perpendicular to each other. PCA, principal component analysis.

The best-fit atomistic structures were analyzed. A visual inspection showed similar looking conformations lying in different orientations in the three-dimensional space, making it difficult to identify common patterns in these. In order to identify the main outcomes in the 100 best-fit structures for each of the four samples, principal component analysis (PCA) was performed on each set of 100 best-fit X-ray models (Fig. 14) (27). PCA shows similarities in protein structures by projecting high-dimensional data onto a few principal components (PCs). The axis on which the *projections* of the original data points show the greatest variance is known as the first PC. The axis along which the data shows the second largest variance is the second PC. When atomistic structures are visualized along a handful of such PCs (usually two or three), similar structures tend to cluster together on the low-dimensional plots. Atomic coordinates were transformed into a small number of directions (PCs) where a given set of structures displayed the largest collective variance (31). Using this method, each set of the 100 best-fit structures of dIgA1 was readily grouped into three PCA groups (green, red and black). For the healthy control, Figure 14 shows the conformer plots for the three PCs (PC1, PC2 and PC3) and the scree plot from the PCA analysis of the 100 best-fit structures. The latter shows that the top three PCs captured 89.4 percent of the total variance in the 100 structures. Each of the three PCA groups revealed a unique class of structure. Images of one representative structure from each PCA group are shown in Figure 15. While the overall angle between the two monomers, shown in *red* and *blue*, appeared similar in all three groups, the main variation is in the position of the Fab arms of the two monomers relative to each other. The scree plots were similar for the four subjects.

To study how the two monomers face each other in the 100 best-fit models, the ‘twist angle’ between the two monomers was calculated using the principal axes of rotation of the Fc region in each monomer. This was done by first constructing a matrix X that is composed of the atomic coordinates of all the α-carbon atoms inside the given atomic cluster with respect to the cluster’s center of mass. Given that all the considered atoms (α-carbon) have an equal mass, the moment of inertia tensor can be written as

(Eq.1) $$I=X^{T}\cdot X$$

**Figure 16:**
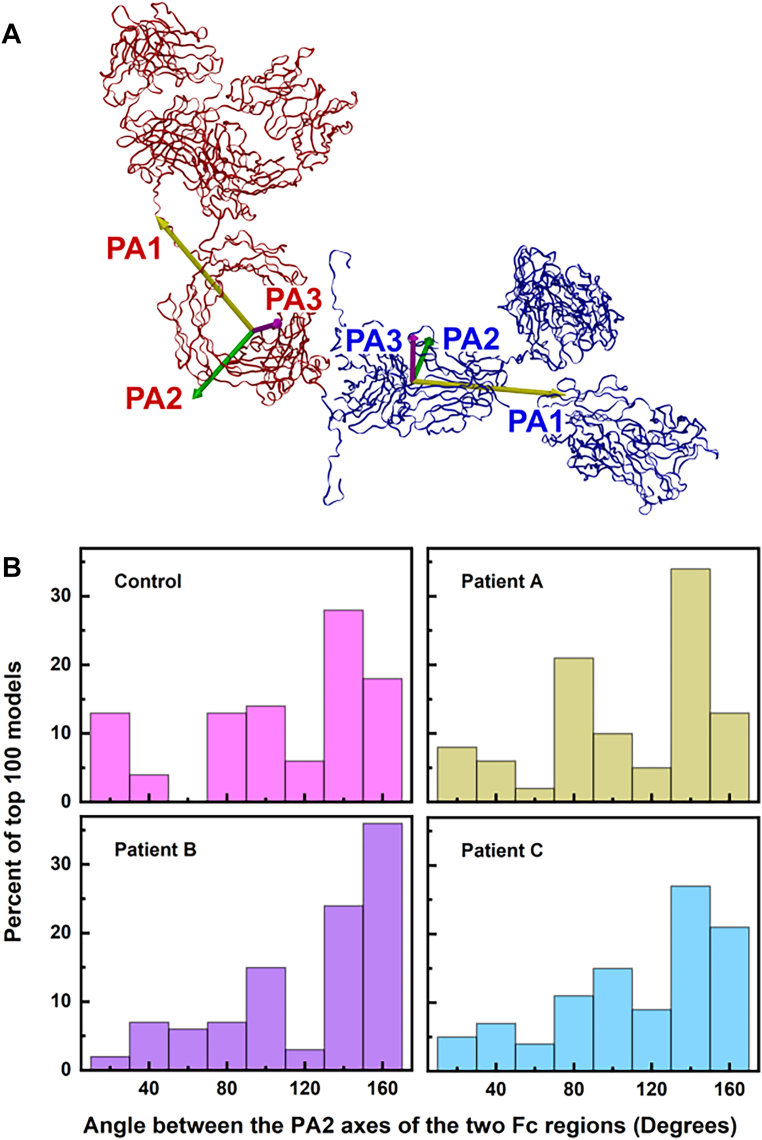
**Twist angles in the top 100 models.***A*, the three principal axes (PA1, PA2, and PA3) calculated for the Fc region of each monomer using the respective moment of inertia tensors. The angle made between the two PA2 axes (*green*) indicates the twist of one monomer with respect to the other. For simplicity of viewing, the glycans have been omitted. *B*, the angle made between the PA2 axes of the Fc regions of the two monomers was calculated for the top 100 models for the four subjects. These were binned in intervals of 20°. An angle of 0° indicates a coplanar conformation, while an angle of 180° indicates a configuration with opposite-facing monomers.

The eigenvectors of I are the principal axes of the given atomic cluster. Figure 16 shows a typical view of the principal axes, denoted as PA1, PA2, and PA3, calculated separately for each of the two Fc regions. The axis of current interest is PA2 (shown in *green*). The angle between the two PA2 vectors is indicative of the amount of ‘twist’ in the configuration of one monomer with respect to the other. When both the monomers are coplanar, *i.e.*, when the two monomers face in the same direction, this angle is zero. A twist angle of 180° means the two monomers are facing opposite to each other. Figure 16 also shows the twist angle calculated for the 100 best-fit models in all the four study subjects, binning them in 20° intervals. Larger twist angles (around 150°) can be seen in all the subjects, although there are also smaller peaks around 90° and 30°. We conclude that the three PCA groups of configurations identified in Figures 14 and 15 give rise to a range of twist angles.

**Figure 17:**
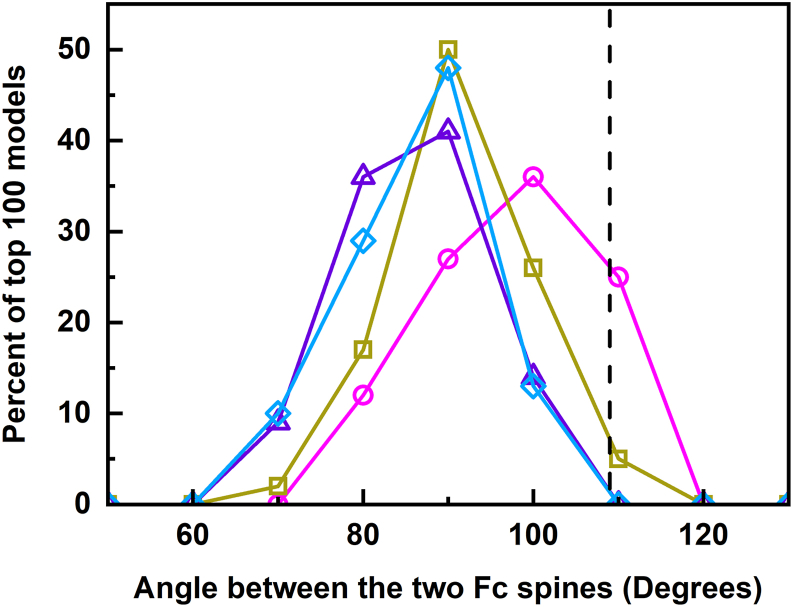
**Intermonomer angles in the top 100 models.** For each of the four subjects, the angles made between the Fc domains of the two IgA monomers were binned in bins of 10 degrees width. A fairly sharp distribution of angles is found, centered at around 90 or 100 degrees (healthy control, ◯; patient A, □; patient B, △; patient C, ◇). The color coding for the four samples is the same as in Figure 11, Figure 12, and 13. The symbols are positioned at the center of each statistical bin. The vertical *dashed line* marks the angle obtained from the IgA1 dimer structure reported in the PDB model 6UE7, this being based on the same definition of the spine axis as used in the present study.

To study the angle between the two IgA1 monomers in dIgA1, the intermonomer angle in each of the 100 best-fit structures was calculated for each of the four study subjects and binned in steps of 10° width in order to obtain a distribution of the angles in the dimer (Fig. 17). Here, the intermonomer angle is defined as that between the Fc domains of the two monomers excluding the Fab arms as they were slightly bent in the original monomer structure used to build the dimer model. This distribution was centered at around 100° for the healthy control and around 90° for patients A, B, and C. For comparison, we also calculated the intermonomer angle in the secretory IgA1 dimer structure containing the J-chain (PDB code 6UE7) that was reported using cryo-electron microscopy (21) but using the same definition of the spine axis of the Fc region as in the present study. This gave 109° and is indicated as the vertical dashed line in Figure 17. This angle is well within the angle distribution for the healthy control, but almost outside the distributions for patients A, B, and C. Thus, there is evidence for different monomer conformations in the dimer structure in the three dIgA1 patients compared to the healthy control.

## Discussion

This comparative study has provided new insights into the molecular structures of the dIgA1 dimer. Often, full-length antibody structures are not crystallizable, thus little is known about their full-length solution structure. Experimentally, the biochemical data showed that, from the immune complex levels, the production of IL-6, the HAA-lectin binding and the *N*- and *O*-glycosylation patterns, distinct differences were seen between the dIgA1 from the healthy control compared to the three patients. Changes in the hinge *O*-glycosylation thus affected IgA–IgG complex formation, the IL6 response, and the HAA lectin binding. It must be acknowledged that the measurement of IgA containing immune complexes was limited to measurement of IgA–IgG complexes. It is clear from mass spectrometry studies that IgA complexes with many other serum proteins in patients with IgAN, including IgA itself, and that these complexes have not been assessed in this study (32). The changes we describe in the structural conformation of dIgA1 in IgAN could conceivably influence binding of proteins other than antiglycan IgG antibodies, and this will be the focus of future studies.

The structural data from AUC, SAXS, and SANS measurements also showed that distinct differences were also seen between the four dIgA1 samples. All four samples were well-behaved in solution and showed stable dimeric structures. The atomistic modeling of the SAXS data based on the sets of best-fit models showed similarities for the four samples. Intriguingly, the analyses of the 100 best-fit structures implied that the relative orientations of the two monomers within the dimer were different in dIgA1 of the healthy control in contrast to those of the three patients (Figure 15, Figure 16, Figure 17). These findings provide new insight into the molecular role of dIgA1 and its association with IgAN.

Taken together, the results show that distinct but correlated differences were often seen between the dIgA1 from the healthy control and the three patients. Thus, dIgA1 HAA lectin binding was significantly higher in two IgAN samples (B, C) than in the healthy control (Fig. 3); dIgA1–IgG complex formation was likewise higher in two IgAN samples (B, C) than in the healthy control (Fig. 4); and the ability to stimulate IL-6 synthesis by human mesangial cells *in vitro* was higher in two IgAN samples (B, C). The X-ray *R*_*g*_ and *R*_*xs−1*_ at zero concentration (Fig. 8) were smaller at 8.62 nm for the IgAN samples (B, C) compared to the other two samples at 8.75 to 8.90 nm. It was noteworthy that the $R_{g}^{0}$ for subjects with high hinge *O*-galactosylation (healthy control and patient A) were significantly higher than subjects with low *O*-galactosylation (patients B and C). The atomistic modeling for 28,023 dIgA1 trial structures all gave good curve fits with the experimental data (Figs. 11 and 12). The outcome of the modeling analyses was similar for the four samples in terms of the twist angles between the two monomers (Fig. 16). However, distinct differences were seen for the intermonomer angles, being slightly larger for the healthy control when compared to the three patient dIgA1 samples (Fig. 17). Overall, it would appear that alterations in the *O*-galactosylation chemical structures at the hinge regions lead to alterations in the dIgA1 structures, with higher *O*-galactosylation being correlated with larger *R*_*g*_ values and altered angles between the two IgA1 monomers in the dimer.

Importantly, inspection of the solution structure of dIgA1 provided insight into the changes seen with the different dIgA1 structures studied here. Up to now, there have been few indications of what makes dIgA1 pathogenic as in IgAN, while the native protein in healthy patients is not pathogenic. Alterations in the *O*-glycosylation of the hinge region have been observed (Table 4), and it is generally accepted that the hinge region in antibodies is conformationally flexible. The structural analyses of the 100 best-fit structures showed that there are changes in the angle between the two Fc spines between the healthy control (peaking at 100°) and the three patients A, B, and C (peaking at 90°). This implies that differences in the hinge *O*-glycosylation can cause alteration in the overall structure, which in turn can cause an increased propensity of the modified IgA molecules to form deposits. These observations imply that the inhibition of the Fc regions to form more compact angles in IgAN may rectify a main cause of IgAN.

The advantage of our combined SAXS-SANS-AUC-MC approach is the opportunity to determine full-length atomistic antibody structures, such as that found in dIgA1. Traditionally solution scattering analyses are at low structural resolutions of around 2 nm, while protein crystallography routinely achieves 10-fold better resolutions. The atomistic modeling of the SAXS and SANS data gives improved analyses compared to previous because known crystal structures are used to constrain the fits of the scattering curves. Our recent studies have resulted in molecular solution structures for all four human IgG subclasses, IgG1-IgG4 (33, 34, 35, 36). The methodology has been recently extended to llama-sourced heavy-chain antibodies, for which a full atomistic structure was determined (36). Interestingly, the methodology is able to detect the effect of deglycosylation on the antibody structure. Changes in the Fc region to become more disorganized in deglycosylated human IgG1 were detectable by the SAXS-SANS-AUC-MC approach (37). Overall, the atomistic modeling approach in combination with high-quality SAXS data with little noise at large *Q* values has been of great value in studying the conformations in antibodies.

## Experimental Procedures

### Purification of human dIgA1

dIgA1 was sourced from three patients with biopsy-proven primary IgAN (patients A, B, and C) and one healthy volunteer (no known kidney disease or acute/chronic illness) that were recruited for the study. The three IgAN patients were recruited based on their known variations in renal histology, risk of clinical progression, and *O*-linked galactosylation of the IgA1 hinge region (Table 1). All three patients were on maximally tolerated doses of renin angiotensin system blockers, and none had previously or were currently receiving immunosuppressive medication. The study was approved by the Leicestershire, Northamptonshire, and Rutland Research Ethics Committee, and written informed consent was obtained from all subjects. Total IgA1 was purified from each serum sample by affinity chromatography using agarose-bound jacalin (Vector Laboratories) (38), before being dialyzed into 137 mM NaCl PBS overnight and concentrated to approximately 2.5 mg/ml with centrifugal filter devices of 50 kDa molecular weight cut-off (Millipore). Purified total IgA1 samples were then either used immediately or stored by freezing at −20 °C until further analysis. A freeze-thaw cycle was limited to a single occasion to ensure sample integrity and minimize protein aggregation.

Purified total IgA1 was separated into its monomer, its dimer, and its high molecular weight immune complexes by size-exclusion chromatography using a Superdex 200 pg column (GE Healthcare Bio-Sciences AB) under the control of an ÄKTA Prime system (Fig. 2*A*). Concentrated dIgA1 samples were treated with Protein G immobilized on cross-linked 4% agarose to remove any IgG contaminants by immunoprecipitation. Immediately prior to structural experiments, each dIgA1 sample was purified on a Superose 6 column (GE Healthcare Bio-Sciences AB) to remove nonspecific aggregates induced through storage and freezing (Fig. 2*B*). Sample purity and integrity were checked using reducing and nonreducing SDS-PAGE before and after data collection (Fig. 2*C*). Samples were dialyzed into PBS buffer (137 mM NaCl, 8.2 mM Na_2_HPO_4_, 2.6 mM KCl, 1.5 mM KH_2_PO_4_, pH 7.4). Prior to structural experiments, dIgA1 samples were stored at 4 °C and were not frozen in order to avoid aggregate formation.

### Activity assays of dIgA1

Primary hMC were used in assays to measure the production of IL-6 induced by incubation with dIgA1. Clonetics Mesangial Cell Systems (Lonza) were maintained in hMC media plus 10% fetal calf serum and gentamicin (Lonza). All hMC cells were used at a passage number lower than 10 and were maintained in a humidified atmosphere of 95% air and 5% CO_2_ at 37 ^o^C. To monitor IgA-induced hMC activation, 50,000 hMC cells were seeded in a 96-well plate and allowed to grow to confluency for 24 h. The cells were serum starved for 24 h before being exposed for 48 h to dIgA1 (50 μg/ml) from each of the donors. At the end of the experiment, tissue culture supernatants were collected and analyzed for their IL-6 concentration by ELISA. The IL-6 levels were normalized against protein content in the lysed cells measured using a Nanodrop spectrometer. Results were from four separate experiments, each performed in triplicate, and data were expressed as fold difference of the IL-6 concentration, analyzed using a one-way ANOVA statistical test.

To measure the formation of IgA–IgG immune complexes, goat anti-human IgA F(ab’)_2_ (Jackson Immunology) at 5 μg/ml in coating buffer (0.05 M carbonate/bicarbonate solution, pH 9.6) was applied to a 96-well immunoplate (Nunc Immunoplate) and incubated at 4 ^o^C for 24 h. After washing, plates were blocked with 2% bovine serum albumin (BSA) in PBS for 1 h at room temperature. After washing the plates, 50 μl of samples diluted 1/500 were applied to duplicate wells and incubated at 4 ^o^C overnight. Following washing, 50 μl of HRP-conjugated rabbit polyclonal anti-IgG (Dako) diluted 1/2000 in PBS was added to each well and incubated for 90 min at room temperature. After washing, o-phenylenediamine dihydrochloride substrate was added to each well. The relative levels of IgA–IgG immune complexes were determined by measuring *A*_492_

### Measurement of the O-linked and N-linked glycosylation of dIgA1

Levels of undergalactosylated dIgA1 were measured using HAA lectin which binds terminal *N*-acetylgalactosamine residues *O*-linked to glycoproteins or glycolipids. To monitor the amount of galactose deficient-IgA (Gd-IgA) in each sample by a lectin binding ELISA, dIgA1 was captured on 96-well immunoplates, coated overnight with 10 mg/ml anti-human IgA antibody (product number A0262, DAKO). Washed and blocked with 2% bovine serum albumin in PBS. Serum samples, diluted 1:100 in PBS, were applied to the plates (5 ml/well) in duplicate and incubated overnight at 4 ^o^C. At this dilution, all wells on the plate were completely saturated with dIgA1. This enables equivalent amounts of dIgA1 from each sample to be tested by the assay. IgA bound to each well was desialylated with 2 units of neuraminidase diluted in 0.5 M sodium acetate buffer (pH 5) with 100 IU/ml penicillin and 100 mg/ml streptomycin, overnight at 37 ^o^C. After washing to remove the neuraminidase, Gd-IgA1 was detected by incubation for 90 min with biotinylated HAA lectin (Sigma L8764) followed by HRP-conjugated avidin (catalogue DY 998, Biotechne). The reaction was developed with o-phenylenediamine dihydrochloride/H_2_O_2_ substrate, and the results read as absorbance at 492 nm. Three standard serum samples with high medium and low HAA lectin binding were included on all plates to allow plate to plate normalization. Results were expressed as *A*_492_. Intraplate and interplate variations were below 10%.

Mass spectrometry was also used to determine the *O*-linked and *N*-linked glycosylation in each subject, using an Impact HD quadrupole time-of-flight MS system (Bruker Daltonics) equipped with a nanoBooster. Ten microgram of dIgA1 from each subject was applied to a 96-well plate, the volume was adjusted to a total of 30 μl, and the dIgA1 was digested with TPCK-treated trypsin after reduction and alkylation to generate glycopeptides for analysis (Table 2). Four microliter of each sample was mixed with 36 μl ultrapure deionized water, and these dilutions were put in the autosampler for the measurement. The obtained raw data were converted to the mzXML format, after which the data were aligned using LacyTools (an in-house developed and online freely available software tool for LC-MS data processing) (https://github.com/Tarskin/LaCyTools) (39) based on seven highly abundant analytes spread over the run time range. An extensive list of previously observed, as well as potential glycoforms, was extracted from the raw data. Glycopeptide data were then curated based on observed mass accuracy (ppm error < |20|), signal to noise >9, and the isotopic pattern quality (less than 20% deviation from the theoretical pattern). A series of glycosylation traits were calculated on the curated data. For the *O*-glycopeptides, the number of *N*-acetylgalactosamines (#N), number of galactoses (#H), number of sialic acids (#S), and the ratios of sialic acid per galactose (SA/H) and galactose per GalNAc (H/N) were calculated. For *N*-glycans, the total level of biantennary and triantennary hybrid type glycans were calculated, as well as the levels of sialylation (S), bisection (B), fucosylation (F), galactosylation (G), and sialic acid per galactose (GS), if applicable.

### AUC sedimentation velocity data and analysis for dIgA1

AUC data were obtained on dIgA1 soon after gel filtration using two Beckman XL-I instruments fitted with AnTi50 and AnTi60 rotors, at speeds of 30,000 and 40,000 rpm at 20 °C (30). The following concentration series in PBS buffer were used: healthy control, 0.45, 0.68, and 0.91 mg/ml; patient A, 0.38, 0.58, and 0.77 mg/ml; patient B, 0.47, 0.70, and 0.94 mg/ml; and patient C, 0.41, 0.61, and 0.81 mg/ml. Sedimentation velocity data (absorbance scans at 280 nm and interference scans) were acquired over 16 h in two sector cells with column heights of 12 mm. All AUC experiments were performed in PBS and repeated in PBS with 100% ^2^H_2_O.

Data were analyzed using SEDFIT (40, 41) based on the continuous *c*(*s*) distribution model with a resolution set as 250, while floating the cell meniscus and bottom and frictional ratio (set at 1.70 as initial run). The partial specific volume $\bar{v}$ was calculated to be 0.724 ml/g from its composition. The buffer density was calculated from its composition using SEDNTERP and was found to be 1.00543 g/ml for PBS in light water and 1.11238 g/ml for PBS in 100% ^2^H_2_O, and the buffer viscosity were taken to be 0.01002 cp. The percentage of dIgA1 species (monomer, dimer, and tetramer) in the total loading concentration was derived using the *c*(*s*) integration function.

### SAXS and SANS data analyses for dIgA1

SAXS data for dIgA1 in PBS buffer in light water were obtained on beamline BM29 at the European Synchrotron Radiation Facility with a ring energy of 6.0 GeV, supplied in seven/eight bunch mode with currents about 194 mA and wavelength 0.09919 nm to reduce the incident X-ray flux (42). The sample-to-detector distance of 2.867 m yielded a scattering vector (*Q*) range from 0.01 nm^−1^ to 4.97 nm^−1^. Samples were contained in water-cooled Perspex cells at 20 °C, of path thickness 1 mm and mica windows of thickness 25 μm. Samples were measured in 10 time frames, each of 1 s, with the samples being moved continuously during beam exposure, these being optimal to eliminate radiation damage effects. Online checks during data acquisition confirmed the absence of radiation damage, after which each set of undamaged frames were averaged. A concentration series, with four concentrations for each sample of dIgA1, was prepared to examine concentration dependence: healthy control—0.33, 0.66, 0.98, and 1.31 mg/ml; patient A—0.31, 0.62, 0.92, and 1.23 mg/ml; patient B—0.32, 0.63, 0.95, and 1.26 mg/ml, and patient C—0.28, 0.57, 0.85, and 1.13 mg/ml. Each experiment was repeated in triplicate to ensure consistency of the results. Hence, a total of 48 datasets with 10 time-frames in each set were acquired to optimize the data collection.

SANS data for dIgA1 were obtained on Instrument LOQ at the pulsed neutron source ISIS, Rutherford Appleton Laboratory (43). Samples were first dialyzed at 6 °C into PBS buffer in 100% heavy water for 36 h with four buffer changes. Samples and buffers were measured in 2 mm thick banjo quartz Hellma cells positioned in a thermostatted rack at 20 °C. The pulsed neutron beam was derived from proton beam currents of approximately 180 μA. Data acquisitions lasted 24 h for dIgA1 samples at concentration of 0.95 mg/ml (healthy subject), 0.71 mg/ml (patient A), 0.99 mg/ml (patient B), and 0.84 mg/ml (patient C). Typically, the dIgA1 neutron *I(Q)* curve contained 61 data points between *Q* values of 0.21 and 2.1 nm^−1^.

The SAXS and SANS curves of *I(Q) versus Q*, where *Q* is the scattering vector and *I(Q)* is the scattering intensity, were used for Guinier analyses in the SCT suite of programs (44). These computed the radius of gyration *R*_*g*_, which is a measure of the elongation of the macromolecule if the internal inhomogeneities of scattering densities has no effect, and the forward scattering intensity at zero scattering angle *I(0)* (45). This was done by identifying a linear region in the plot of ln *I(Q) versus Q*^2^ at low *Q* values, in the *Q*.*R*_*g*_ range up to 1.6:

(Eq. 2) $$\ln\;I\left(Q\right)=\ln\;I\left(0\right)-\frac{{R_{g}}^{2}Q^{2}}{3}$$

For an elongated protein such as dIgA1, the mean cross-sectional radius of gyration *R*_*xs*_ is obtained from plots of ln *I(Q)*.*Q* against $Q^{2}$ in a larger *Q* range than that used for calculating *R*_*g*_, and this region of the scattering curve is generally flatter than that for the *R*_*g*_. For immunoglobulins, it is known that the cross-sectional plot exhibits two regions, a steeper innermost one and a flatter outermost one (46), and the two analyses are denoted by *R*_*xs−1*_ and *R*_*xs−2*_, respectively. The *R*_*xs−1*_ parameter represents the averaged overall spatial separation of the Fab and Fc regions, whereas the *R*_*xs−2*_ parameter represents the averaged spatial cross section of the two Fab and one Fc region.

(Eq. 3) $$\ln\left[I\left(Q\right)Q\right]={\left[I\left(Q\right)Q\right]}_{Q\to 0}-\frac{{R_{xs}}^{2}Q^{2}}{2}$$

The indirect Fourier transformation of the full scattering curve *I(Q)* in reciprocal space into real space gives the distance distribution function *P(r)*, and this was computed using the program GNOM (47, 48):

(Eq. 4) $$P\left(r\right)=\frac{1}{2\pi^{2}}\int_{0}^{\infty }I\left(Q\right)Qr\;\sin\left(Qr\right)dQ$$

The *P*(*r*) curve denotes the distribution of all interatomic distances r between all volume elements in the macromolecule. The tail of the *P*(*r*) curve gives *L* as the largest dimension for the macromolecule and the peaks *M1* and *M2* as the most common interatomic distance vectors, as well as providing an alternative way of calculating *R*_*g*_.

### Atomistic modeling of dIgA1

To initiate the dIgA1 modeling, the atomistic structure of the full-length glycosylated IgA1 monomer was taken from the earlier SAXS and SANS curve modeling using molecular dynamics and Monte Carlo methods (27). This structure showed a slightly different orientation of the monomer tailpieces than the original coarse-grained IgA1 structure (26). In order to create a starting dimer model, the first step was to define the Cartesian coordinate system. The *x*-axis was defined such that it ran through the middle of the Fc region of the monomer, while the origin, (*x*, *y*, *z*) = (0, 0, 0), was placed a small distance below the Fc fragment. The *y*−*z* plane cuts perpendicularly through the x-axis at the origin (Fig. 7). The dimer IgA1 molecule was computationally generated by creating a copy of the monomer about the *y*−*z* plane, so that the Fc regions of the two monomers are coaxial on the *x*-axis.

Eleven starting models for dIgA1 were created. The J chain was disregarded in these models for reason of simplicity. In the first three models, the monomer was duplicated coaxially on the other side of the *y*−*z* plane, and the monomers were shifted along the *x*-axis such that the intermonomer distance was 1.4 nm, 2.78 nm, and 4.6 nm, respectively, between the bases of the two Fc regions on the *x*-axis. These values were chosen to ensure comparability with our 2008 study (26). In the remaining eight starting models, the monomers were separated by 1.4 nm but were also displaced from each other by 2.0 nm and 4.0 nm in both the y and z axes, in positive and negative directions. These 11 starting models will hereafter be referred to as models 14, 278, 46, y20, y-20, y40, y-40, z20, z-20, z40, and z-40. The starting models showed close agreement with the subsequently published cryo-EM structure of the dimer of the IgA1 Fc region in which the J chain acted as a spacer between the two Fc regions (23).

Using the same basic protocol used by Bonner *et al.* (22), each of these starting monomers then had their second monomer rotated in such a way as to explore all of the space on that half of the model. To do this, a Python module named molecuPy was created, which is now publicly available, but renamed as atomium (49). To rotate a monomer by an angle θ about the origin, this module uses the standard rotation matrices to perform the atomic coordinate transformation and modifies the given PDB file in-place:

$$R_{x}\left(\theta \right)=\left[\begin{array}{ccc} 1 & 0 & 0\\ 0 & \cos\;\theta & -\sin\;\theta \\ 0 & \sin\;\theta & \cos\;\theta \end{array}\right]$$

(Eq. 5) $$R_{y}\left(\theta \right)=\left[\begin{array}{ccc} \cos\;\theta & 0 & \sin\;\theta \\ 0 & 1 & 0\\ -\sin\;\theta & 0 & \cos\;\theta \end{array}\right]$$

$$R_{z}\left(\theta \right)=\left[\begin{array}{ccc} \cos\;\theta & -\sin\;\theta & 0\\ \sin\;\theta & \cos\;\theta & 0\\ 0 & 0 & 1 \end{array}\right]$$

For instance, if a rotation of a molecule about the *x*-axis is to be performed by an angle θ, the new atomic coordinates after the rotation, $r_{\text{new}}$, are obtained from the old coordinates, $r_{\text{old}}$, by multiplying the latter by the matrix $R_{x}\left(\theta \right)$:

(Eq. 6) $${r_{\text{new}}=R}_{x}\left(\theta \right)r_{\text{old}}$$

By rotating one of the IgA1 monomers in increments of 10° about each of the *x*, y, and *z* axes, in a range from −90° to 90° and holding the other IgA1 monomer fixed in position, the entire three-dimensional space on one side of the dimer was probed. This produced 19^3^ = 6859 atomistic dimer structures for each starting model, making a total of 75,449 trial dimer models. Many of the dIgA1 structures showed atomistic steric clashes between the two monomers which were physically unrealistic. These overlapping structures were identified using a Python script and were discarded. At the end of this process, 28,023 structures of the IgA1 dimer showed no steric clashes, and only these were used for further analysis.

To determine which of the 28,023 trial atomistic dimer structures best fitted the experimental scattering data, the software suite SASSIE-web was used (25). Within SASSIE-web, the SasCalc module was used to calculate the theoretical scattering curves corresponding to each of the 28,023 dimer structures. The SasCalc module applies the golden ratio method to calculate the scattering intensity *I*(*Q*) of an atomistic structure as a function of the scattering vector *Q* (50). The computed scattering curves were compared against the experimental scattering curves, extrapolated to zero concentration, for the four study subjects (healthy control and patients A, B, and C). The goodness-of-fit for each computational structure was calculated using the *R*-factor, defined as

(Eq. 7) $$R=\frac{\sum \left|\left|I_{\text{Expt}}\left(Q\right)\right|-\eta \left|I_{\text{Theor}}\left(Q\right)\right|\right|}{\sum \left|I_{\text{Expt}}\left(Q\right)\right|}$$

where $I_{\text{Expt}}\left(Q\right)$ and $I_{\text{Theor}}\left(Q\right)$ are the experimental and theoretically calculated scattered intensities, while η is a scaling factor that is used to match the theoretical and experimental scattering intensities at *Q* = 0. Principal component analysis using the Bio3D package (51, 52) identified four distinct clusters of full-length dIgA1 structures. Artwork (Figs. 15 and 16) was drawn using VMD (53).

## Data availability

All data are contained within this manuscript. The 100 and single best-fit models for dIgA1 that correspond to the X-ray fit searches (Fig. 11 and 12) are available in Supporting Information. The single best-fit dIgA1 models were also deposited in the SASBDB database (https://www.sasbdb.org/) (54) with the reference codes SASDYW2 (healthy control), SASDYX2 (patient A), SASDYY2 (patient B), and SASDYZ2 (patient C).

## Supporting information

This article contains supporting information.

## Conflict of interest

The authors declare that they have no conflicts of interest with the contents of this article.
